# Degeneracy in the robust expression of spectral selectivity, subthreshold oscillations, and intrinsic excitability of entorhinal stellate cells

**DOI:** 10.1152/jn.00136.2018

**Published:** 2018-05-02

**Authors:** Divyansh Mittal, Rishikesh Narayanan

**Affiliations:** Cellular Neurophysiology Laboratory, Molecular Biophysics Unit, Indian Institute of Science, Bangalore, India

**Keywords:** coincidence detection, gamma frequency, heterogeneity, membrane potential oscillations, membrane resonance, spike-triggered average

## Abstract

Biological heterogeneities are ubiquitous and play critical roles in the emergence of physiology at multiple scales. Although neurons in layer II (LII) of the medial entorhinal cortex (MEC) express heterogeneities in channel properties, the impact of such heterogeneities on the robustness of their cellular-scale physiology has not been assessed. Here, we performed a 55-parameter stochastic search spanning nine voltage- or calcium-activated channels to assess the impact of channel heterogeneities on the concomitant emergence of 10 in vitro electrophysiological characteristics of LII stellate cells (SCs). We generated 150,000 models and found a heterogeneous subpopulation of 449 valid models to robustly match all electrophysiological signatures. We employed this heterogeneous population to demonstrate the emergence of cellular-scale degeneracy in SCs, whereby disparate parametric combinations expressing weak pairwise correlations resulted in similar models. We then assessed the impact of virtually knocking out each channel from all valid models and demonstrate that the mapping between channels and measurements was many-to-many, a critical requirement for the expression of degeneracy. Finally, we quantitatively predict that the spike-triggered average of SCs should be endowed with theta-frequency spectral selectivity and coincidence detection capabilities in the fast gamma-band. We postulate this fast gamma-band coincidence detection as an instance of cellular-scale-efficient coding, whereby SC response characteristics match the dominant oscillatory signals in LII MEC. The heterogeneous population of valid SC models built here unveils the robust emergence of cellular-scale physiology despite significant channel heterogeneities, and forms an efficacious substrate for evaluating the impact of biological heterogeneities on entorhinal network function.

**NEW & NOTEWORTHY** We assessed the impact of heterogeneities in channel properties on the robustness of cellular-scale physiology of medial entorhinal cortical stellate neurons. We demonstrate that neuronal models with disparate channel combinations were endowed with similar physiological characteristics, as a consequence of the many-to-many mapping between channel properties and the physiological characteristics that they modulate. We predict that the spike-triggered average of stellate cells should be endowed with theta-frequency spectral selectivity and fast gamma-band coincidence detection capabilities.

## INTRODUCTION

Networks in the nervous system are endowed with several forms of heterogeneities, which are known to play vital roles in the emergence of physiology and behavior. These ubiquitous forms of heterogeneities have been shown to either aid or hamper physiology in a manner that is reliant on several variables, including the system under consideration, its specific function, and the state of the system. Such state-dependent impact of biological heterogeneities has necessitated system- and state-dependent quantitative analyses in assessing the precise role of these heterogeneities in specific neuronal structures and associated emergent functions (Angelo et al. 2012; Anirudhan and Narayanan 2015; Cadwell et al. 2016; Chelaru and Dragoi 2008; Das et al. 2017; Ecker et al. 2011; Fuzik et al. 2016; Gjorgjieva et al. 2016; Goaillard et al. 2009; Grashow et al. 2010; Kohn et al. 2016; Marder 2011; Marder and Goaillard 2006; Marder et al. 2014; Marder and Taylor 2011; Mukunda and Narayanan 2017; Nusser 2009; Padmanabhan and Urban 2010; Prinz et al. 2004; Rathour and Narayanan 2014; 2012; Renart et al. 2003; Shamir and Sompolinsky 2006; Srikanth and Narayanan 2015; Tikidji-Hamburyan et al. 2015; Tripathy et al. 2013; Voliotis et al. 2014; Wang and Buzsáki 1996; Zhou et al. 2013).

Neurons in layer II (LII) of the rodent medial entorhinal cortex (MEC) have been implicated in spatial navigation, especially since the cells in LII MEC are known to act as grid cells that generate action potentials in a gridlike pattern as the animal traverses an arena (Buzsáki and Moser 2013; Hafting et al. 2005; Moser et al. 2008, 2014, 2015; Ray et al. 2014; Tang et al. 2014). Ever since the discovery of grid cells, several theoretical and computational models have been proposed for their emergence, and have been tested from several different perspectives with varying degrees of success (Burak and Fiete 2006, 2009; Burgess et al. 2007; Bush and Burgess 2014; Couey et al. 2013; Domnisoru et al. 2013; Giocomo et al. 2011b; Jeewajee et al. 2008; Navratilova et al. 2012; O’Keefe and Burgess 2005; Rowland et al. 2016; Schmidt-Hieber and Häusser 2013; Schmidt-Hieber et al. 2017; Sreenivasan and Fiete 2011; Welinder et al. 2008; Yoon et al. 2013). Although these models and associated experiments have provided significant insights into entorhinal function, a lacuna common to these models relates to the systematic assessment of the impact of the different biological heterogeneities in the medial entorhinal cortex. Specifically, a systematic evaluation of the role of different forms of network heterogeneities, including those in channels, structural, intrinsic and synaptic properties and in afferent connectivity, with reference to entorhinal physiology has been lacking.

A first and essential step in addressing these and other related questions on the impact of biological heterogeneities on entorhinal network function is to assess the robustness of cellular physiology in the presence of well-established heterogeneities in channel properties. Specifically, measurements of channel properties, including kinetics, voltage-dependent gating, and conductance values, from entorhinal neurons are known to exhibit significant variability across neurons (Bruehl and Wadman 1999; Castelli and Magistretti 2006; Dickson et al. 2000; Dudman and Nolan 2009; Fransén et al. 2004; Magistretti and Alonso 1999; Pastoll et al. 2012; Schmidt-Hieber and Häusser 2013). Despite this, entorhinal neurons exhibit signature in vitro electrophysiological characteristics that robustly fall into specific ranges for each physiologically relevant measurement. How do these neurons achieve such cellular-scale robustness in their physiology despite widely variable conductances and channel properties? Is there a requirement for individual channels or pairs of channels to be maintained at specific levels with specific properties for signature in vitro electrophysiological properties to emerge? Is there a one-to-one mapping between individual channels and the physiological properties that they regulate?

In this study, we build a conductance-based intrinsically heterogeneous population of LII stellate cell (SC) models of the medial entorhinal cortex that satisfied several of their unique in vitro electrophysiological signatures. We employed this heterogeneous population of LII SC models to demonstrate the expression of cellular-scale degeneracy (Edelman and Gally 2001) in the concomitant emergence of these measurements. Specifically, we showed that LII SC with very similar electrophysiological characteristics emerged from disparate channel and parametric combinations. We employed these models to demonstrate the differential and variable dependencies of measurements on underlying channels. Our observations also showed that the mapping between channels and measurements was many-to-many, whereby individual channels contributed to several measurements and individual measurements were dependent on several channels. Finally, we employed this electrophysiologically validated model population to make quantitative testable predictions that the spike-triggered average (STA) of LII SCs should be endowed with theta-frequency spectral selectivity and coincidence detection capabilities in the fast gamma-band. We postulate this fast gamma-band coincidence detection to be an instance of cellular-scale efficient coding (Narayanan and Johnston 2012), whereby the response properties of the neuron match the dominant oscillatory band in the superficial layers of MEC (Colgin 2016; Colgin et al. 2009; Colgin and Moser 2010; Trimper et al. 2017). The heterogeneous population of models built here also forms an efficacious substrate for constructing network models of the entorhinal cortex, toward assessing the impact of cellular and channel properties and associated heterogeneities on emergent behavior such as grid cell formation.

## METHODS

**Fig. 1.**
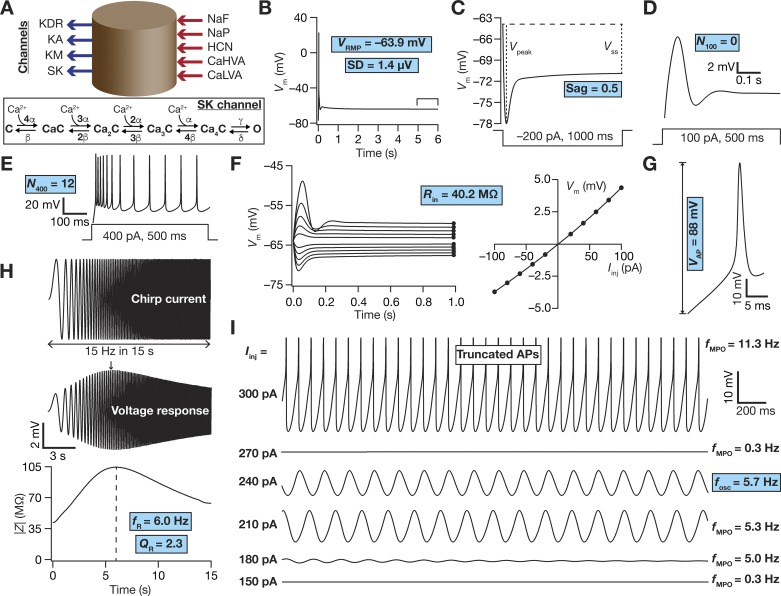
Base model and measurements. *A*: schematic representation of a single-compartment model for medial entorhinal cortex (MEC) layer II stellate cell specifying inward (inward arrows) and outward (outward arrows) currents. *Inset*: 6-state kinetic model of SK channels. Parametric values were α = 10 µM/s, β = 0.5/s, γ = 600/s, and δ = 400/s. *B–I*: the 10 physiologically relevant measurements (highlighted in cyan) used to characterize stellate cells. *B*: resting membrane potential (*V*_RMP_) and its standard deviation (SD) were computed by taking the mean and SD, respectively, of the membrane potential (*V*_m_) between 5- and 6-s duration (window specified in the figure) when no current was injected. All the other measurements were performed after the model settled at its *V*_RMP_ at 6 s. *C*: Sag ratio (Sag) was measured as the ratio of the steady-state membrane potential deflection (*V*_SS_) to peak membrane potential deflection (*V*_peak_) in the voltage response of the model to a hyperpolarizing step current of 200 pA for a duration of 1,000 ms. *D* and *E*: voltage response of the model to a step current of 100 pA (*D*) or 400 pA (*E*) for a stimulus duration of 500 ms was used to measure the number of action potentials (*N*_100_ or *N*_400_) elicited for the respective current injection. *F*: input resistance (*R*_in_) computation. *F*, *left*: 1,000-ms-long step currents from −100 pA to 100 pA were injected into the cell in steps of 20 pA to record the steady-state voltage response (black circles at the end of each trace). *F, right*: steady-state voltage response vs. injected current (*V–I*) plot obtained from the traces on the left panel. The slope of a linear fit to the *V–I* plot defined *R*_in_. *G*: amplitude of action potential (*V*_AP_) was measured as the difference between the peak voltage achieved during an action potential and *V*_RMP_. *H*: impedance-based measurements. *H, top*: Chirp current stimulus injected into the cell. *H, middle*: voltage response of the model to chirp stimulus injection. The arrow depicts the location of the maximal response. *H, bottom*: impedance amplitude profile showing the resonance frequency (*f*_R_) at which the model elicited peak response and resonance strength (*Q*_R_), the ratio of impedance amplitude at *f*_R_ to impedance amplitude at 0.5 Hz. *I*: membrane potential oscillations (MPOs). Shown are representative voltage traces (3-s duration) for different depolarizing current injections (*I*_inj_). The emergence of subthreshold oscillations in the theta range may be observed in traces at intermediate values of *I*_inj_, with the model switching to action potential firing at higher *I*_inj_. The frequency of subthreshold oscillations measured at a perithreshold voltage was defined as *f*_osc_, whereas the frequency of membrane potential oscillations obtained with other *I*_inj_ was represented by *f*_MPO_.

We employed a single-compartmental cylinder model of 70-μm diameter and 75-μm length (Fig. 1*A*). The choice of a single-compartmental model was largely driven by the absence of direct and detailed electrophysiological characterization of dendritic intrinsic properties or of ion channels that express in LII SCs. As a consequence, morphologically precise models with specific channel expression profiles and matched intrinsic properties were infeasible. On the other hand, as the somatic channel properties and intrinsic physiological measurements of LII SCs are well characterized, we employed a single-compartmental model that would not have to make explicit or implicit assumptions about dendritic physiology. Additionally, as a goal of this study was to develop an intrinsic heterogeneous model population of LII SCs toward their incorporation into network models, it was essential to ensure that the computational complexity of single neurons was minimal. A single-compartmental conductance-based model population that was endowed with the different ion channels and systematically reflects intrinsic heterogeneities in LII SCs served as an efficacious means to achieve this goal as well.

### Passive and Active Neuronal Properties

Passive properties were incorporated into the model as an RC circuit that was defined through a specific membrane resistance, *R*_m_, and a specific membrane capacitance, *C*_m_. We introduced nine different active channel conductances into the model (Fig. 1*A*): fast sodium (NaF), delayed rectifier potassium (KDR), hyperpolarization-activated cyclic-nucleotide gated (HCN) nonspecific cationic, persistent sodium (NaP), A-type potassium (KA), low-voltage-activated calcium (LVA), high-voltage-activated calcium (HVA), M-type potassium (KM), and small-conductance calcium-gated potassium (SK) channels. The channel kinetics and voltage dependencies for NaF, KDR, and KA were from Dudman and Nolan (2009); for HCN from Dickson et al. (2000), Fransén et al. (2004), and Schmidt-Hieber and Häusser (2013); for NaP from Dickson et al. (2000), Fransén et al. (2004), and Magistretti and Alonso (1999); for HVA from Bruehl and Wadman (1999) and Castelli and Magistretti (2006); for LVA from Bruehl and Wadman (1999) and Pastoll et al. (2012); for SK from Sah and Clements (1999) and Sah and Isaacson (1995); and for KM from Shah et al. (2008).

All channel models were based on Hodgkin-Huxley formulation (Hodgkin and Huxley 1952) except for the SK channel, which was modeled using a six-state Markovian kinetics model (Fig. 1*A*). Sodium, potassium, and HCN channel currents were modeled using the Ohmic formulation and calcium channels followed the Goldman-Hodgkin-Katz (GHK) formulation (Goldman 1943; Hodgkin and Katz 1949) for current computation. The reversal potentials for Na^+^, K^+^, and HCN channel were 50, −90, and −20 mV, respectively. Calcium current through voltage-gated calcium channels contributed to cytosolic calcium concentration ([Ca]*_c_*), and its decay was defined through simple first-order kinetics (Carnevale and Hines 2006; Destexhe et al. 1993; Honnuraiah and Narayanan 2013; Narayanan and Johnston 2010; Poirazi et al. 2003):

$$\frac{\text{d}{\left[\text{Ca}\right]}_{c}}{\text{d}t}=-\frac{10,000I_{Ca}}{36\cdot dpt\cdot F}+\frac{{\left[\text{Ca}\right]}_{\infty }-{\left[\text{Ca}\right]}_{c}}{\tau_{Ca}}$$

where *F* represented Faraday’s constant, τ_ca_ defined the calcium decay time constant, *dpt* = 0.1 μm was the depth of the shell for cytosolic calcium dynamics, and [Ca]_∞_ = 100 nM was the steady-state value of the [Ca]*_c_*.

Channel models were directly adopted in cases where they were explicitly based on direct measurements from LII SC channels. In cases where such explicit models were not available, model formulations were taken from other cell types and were explicitly adapted to match direct in vitro electrophysiological measurements. As channel models were either adopted from different studies or were adapted to match experimental observation, in what follows, we provide details of the models that we employed for gating and kinetics of each channel. The parameters that define these channel models are described in Table 1, along with the base values of the 55 passive and active parameters that govern these models. The values of parameters governing kinetics (e.g., activation time constants) and regulating gating properties (e.g., half-maximal activation voltages) were not changed from their respective default values (derived from corresponding electrophysiological recordings) in hand tuning the base model. The tuning process that resulted in the base model (Fig. 1, Table 1) involved adjustment of conductances associated with each of the different channels toward matching with signature electrophysiological characteristics of SCs (listed in Table 2).

**Table 1.** Base value and range of parameters used in generating the model population

No.	Parameter (Unit)	Description	Base	Min	Max
*Fast sodium (NaF) channel*					
*1*	*g^NaF^* (mS/cm^2^)	Maximal conductance of NaF	4.2	2.1	8.5
*2*	$V_{m}^{NaF}$ (mV)	Half-maximal voltage of activation of NaF	−26.1	−31.1	−21.1
*3*	$k_{m}^{NaF}$ (mV)	Slope of activation of NaF	9.38	7.51	11.26
*4*	$F_{m}^{NaF}$	Scaling factor for activation time constant of NaF	1	0.8	1.2
*5*	$V_{h}^{NaF}$ (mV)	Half-maximal voltage of inactivation of NaF	−23.8	−28.8	−18.8
*6*	$k_{h}^{NaF}$ (mV)	Slope of inactivation of NaF	6.1	4.9	7.3
*7*	$F_{h}^{NaF}$	Scaling factor for inactivation time constant of NaF	1	0.8	1.2
*Delayed rectifier potassium (KDR) channel*					
*8*	*g^KDR^* (mS/cm^2^)	Maximal conductance of KDR	3.2	1.5	6.4
*9*	$V_{n}^{KDR}$ (mV)	Half-maximal voltage of activation of KDR	−17.6	−22.6	−12.6
*10*	$k_{n}^{KDR}$ (mV)	Slope of activation of KDR	19.6	15.7	23.6
*11*	$F_{n}^{KDR}$	Scaling factor for activation time constant of KDR	1	0.8	1.2
*Hyperpolarization-activated cyclic-nucleotide-gated (HCN) channel*					
*12*	*g^HCN^* (µS/cm^2^)	Maximal conductance of slow HCN	33.3	16	67
*13*	$HCN_{ms}^{mf}$	Ratio of fast to slow HCN maximal conductance	1.85	1.5	2.2
*14*	$V_{mf}^{HCN}$ (mV)	Half-maximal voltage of activation of fast HCN	74.2	69.2	79.2
*15*	$V_{ms}^{HCN}$ (mV)	Half-maximal voltage of activation of slow HCN	2.83	−2.17	7.83
*16*	$k_{mf}^{HCN}$ (mV)	Slope of activation of fast HCN	9.78	7.8	11.7
*17*	$k_{ms}^{HCN}$ (mV)	Slope of activation of slow HCN	15.9	12.7	19.1
*18*	$F_{mf}^{HCN}$	Scaling factor for activation time constant of fast HCN	1	0.8	1.2
*19*	$F_{ms}^{HCN}$	Scaling factor for activation time constant of slow HCN	1	0.8	1.2
*Persistent sodium (NaP) channel*					
*20*	*g^NaP^* (µS/cm^2^)	Maximal conductance of NaP	34	17	68
*21*	$V_{m}^{NaP}$ (mV)	Half-maximal voltage of activation of NaP	48.7	43.7	53.7
*22*	$k_{m}^{NaP}$ (mV)	Slope of activation of NaP	4.4	3.52	5.28
*23*	$F_{m}^{NaP}$	Scaling factor for activation time constant of NaP	1	0.8	1.2
*24*	$V_{h}^{NaP}$ (mV)	Half-maximal voltage of inactivation of NaP	48.8	43.8	53.8
*25*	$k_{h}^{NaP}$ (mV)	Slope of inactivation of NaP	9.9	7.9	11.9
*26*	$F_{h}^{NaP}$	Scaling factor for inactivation time constant of NaP	1	0.8	1.2
*A-type potassium (KA) channel*					
*27*	*g^KA^* (µS/cm^2^)	Maximal conductance of KA	25	12.5	50
*28*	$V_{m}^{KA}$ (mV)	Half-maximal voltage of activation of KA	−18.3	−23.3	−13.3
*29*	$k_{m}^{KA}$ (mV)	Slope of activation of KA	15	12	18
*30*	$F_{m}^{KA}$	Scaling factor for activation time constant of KA	1	0.8	1.2
*31*	$V_{h}^{KA}$ (mV)	Half-maximal voltage of inactivation of KA	−58	−63	−53
*32*	$k_{h}^{KA}$ (mV)	Slope of inactivation of KA	8.2	6.6	9.8
*33*	$F_{h}^{KA}$	Scaling factor for inactivation time constant of KA	1	0.8	1.2
*High-voltage-activated (HVA) calcium channel*					
*34*	*g^HVA^*, (mS/cm^2^)	Maximal conductance of HVA	0.18	0.09	0.36
*35*	$V_{m}^{HVA}$ (mV)	Half-maximal voltage of activation of HVA	11.1	6.1	16.1
*36*	$k_{m}^{HVA}$ (mV)	Slope of activation of HVA	8.4	6.7	10.0
*37*	$F_{m}^{HVA}$	Scaling factor for activation time constant of HVA	1	0.8	1.2
*38*	$V_{h}^{HVA}$ (mV)	Half-maximal voltage of inactivation of HVA	37	32	42
*39*	$k_{h}^{HVA}$ (mV)	Slope of inactivation of HVA	9	7.2	10.8
*40*	$F_{h}^{HVA}$	Scaling factor for inactivation time constant of HVA	1	0.8	1.2
*Low-voltage-activated (LVA) calcium channel*					
*41*	*g^LVA^* (µS/cm^2^)	Maximal conductance of LVA	90	41.9	167.6
*42*	$V_{m}^{LVA}$ (mV)	Half-maximal voltage of activation of LVA	−52.4	−57.4	−47.4
*43*	$k_{m}^{LVA}$ (mV)	Slope of activation of LVA	8.2	6.5	9.8
*44*	$F_{m}^{LVA}$	Scaling factor for activation time constant LVA	1	0.8	1.2
*45*	$V_{h}^{LVA}$ (mV)	Half-maximal voltage of inactivation of LVA	−88.2	−93.2	−83.2
*46*	$k_{h}^{LVA}$ (mV)	Slope of inactivation of LVA	6.67	5.34	8.01
*47*	$F_{h}^{LVA}$	Scaling factor for inactivation time constant of LVA	1	0.8	1.2
*M-type potassium (KM) channel*					
*48*	*g^KM^* (mS/cm^2^)	Maximal conductance of KM	0.12	0.06	0.25
*49*	$V_{m}^{KM}$ (mV)	Half-maximal voltage of activation of KM	−40	−45	−35
*50*	$k_{m}^{KM}$ (mV)	Slope of activation of KM	−10	−8	−12
*51*	$F_{m}^{KM}$	Scaling factor for activation time constant of KM	1	0.8	1.2
*Small-conductance calcium-activated potassium (SK) channel*					
*52*	*g^SK^* (µS/cm^2^)	Maximal conductance of SK	52	26	104
*Passive properties and cytosolic calcium handling*					
*53*	*R_m_* (kΩ cm^2^)	Specific membrane resistance	40	20	80
*54*	τ*_Ca_* (ms)	Time constant of cytosolic calcium decay	78	39	156
*55*	*C_m_* (µF/cm^2^)	Specific membrane capacitance	1	0.75	1.25

Whereas conductance values were scaled from 0.5 × to 2 × , scaling factors for time constants were set in the range 0.8 × to 1.2 × , the half-maximal voltages were shifted by 5 mV on either side of their default values, and the slope of the sigmoidal activation/inactivation curves were scaled by 20% on either side of the respective default values. For parameters other than conductance values, these ranges were chosen to match with respective experimental variability.

**Table 2.** Physiologically relevant range of LII stellate cell measurements

No.	Intrinsic Measurement (Unit)	Valid Range
*1*	Resting membrane potential, *V*_RMP_ (mV)	−65 to −60
*2*	SD of membrane potential (resting), SD (mV)	<0.01
*3*	Sag ratio	0.35–0.65
*4*	Input resistance, *R*_in_ (MΩ)	35–65
*5*	Resonance strength, *Q*_R_	<3.5
*6*	Resonance frequency, *f*_R_ (Hz)	3–12
*7*	Perithreshold MPO frequency, *f*_osc_ (Hz)	3–12
*8*	No. of APs for a 100-pA step current for 500 ms, *N*_100_	0
*9*	No. of APs for a 400-pA step current for 500 ms, *N*_400_	7–16
*10*	AP amplitude, *V*_AP_, (mV)	>75

Experimental bounds on each intrinsic measurement involved in the validation process of stochastically generated models. Although the constraint on the SD of resting membrane potential ensures that there are no membrane potential oscillations at rest, the rest of the bounds were derived from previous electrophysiological measurements (Boehlen et al. 2013; Pastoll et al. 2012).

In channels that employed the Hodgkin-Huxley formulation, the model evolved by modifications to one or two gating particles, with each gating particle following first-order kinetics as follows:

$$\frac{\text{d}m}{\text{d}t}=\frac{m_{\infty }-m}{\tau_{m}}$$

where *m*_∞_ and τ*_m_*, respectively, defined the steady-state value and the time constant of the state variable that governed the gating particle. Channel gating and kinetics were appropriately adjusted for temperature dependence from corresponding experimental measurements.

#### The fast sodium channel.

The NaF model was adopted from Dudman and Nolan (2009), and the current through this sodium channel was:

$$I_{NaF}=g^{NaF}m^{3}h(V-E_{Na}).$$

The activation gating particle was defined by:

$$m_{\infty }^{NaF}={\left[1+\exp\left(\frac{V_{m}^{NaF}-V}{k_{m}^{NaF}}\right)\right]}^{-1}\;\tau_{m}^{NaF}=F_{m}^{NaF}\left(\frac{1}{\alpha_{m}^{NaF}+\beta_{m}^{NaF}}\right)$$

$$\alpha_{m}^{NaF}=\frac{4\left[\left(V+33\right)/9\right]}{\left\{1-\exp\left[-\left(V+33\right)/9\right]\right\}}\;\beta_{m}^{NaF}=\frac{27.6\left[\left(V+58\right)/-12\right]}{\left\{1-\exp\left[\left(V+58\right)/12\right]\right\}}.$$

The inactivation gating particle was defined by:

$$h_{\infty }^{NaF}=1-{\left[1+\exp\left(\frac{V_{h}^{NaF}-V}{k_{h}^{NaF}}\right)\right]}^{-1}\;\tau_{h}^{NaF}=F_{h}^{NaF}\left(\frac{1}{\alpha_{h}^{NaF}+\beta_{h}^{NaF}}\right)$$

$$\alpha_{h}^{NaF}=\frac{0.36\left[\left(V+48\right)/-12\right]}{\left\{1-\exp\left[\left(V+48\right)/12\right]\right\}}\;\beta_{h}^{NaF}=\frac{0.4\left[\left(V+11\right)/6\right]}{\left\{1-\exp\left[-\left(V+11\right)/6\right]\right\}}.$$

#### The delayed rectifier potassium channel.

The KDR model was adopted from Dudman and Nolan (2009), and the current through this potassium channel was:

$$I_{KDR}=g^{KDR}n^{4}\left(V-E_{K}\right).$$

The activation gating particle was governed by the following equations:

$$n_{\infty }^{KDR}={\left[1+\exp\left(\frac{V_{n}^{KDR}-V}{k_{n}^{KDR}}\right)\right]}^{-1}\;\tau_{n}^{KDR}=F_{n}^{KDR}\left(\frac{1}{\alpha_{n}^{KDR}+\beta_{n}^{KDR}}\right)$$

$$\alpha_{n}^{KDR}=\frac{0.2\left[\left(V+38\right)/10\right]}{\left\{1-\exp\left[-\left(V+38\right)/10\right]\right\}}\;\beta_{n}^{KDR}=\frac{0.6294\left[\left(V+47\right)/-35\right]}{\left\{1-\exp\left[\left(V+47\right)/35\right]\right\}}.$$

#### The hyperpolarization-activated cyclic-nucleotide-gated channel.

The HCN channel model was adopted from Dickson et al. (2000), Fransén et al. (2004), and Schmidt-Hieber and Häusser (2013) and the current through this nonspecific cationic channel was:

$$I_{h}=g^{HCN}\left(ms^{HCN}+HCN_{ms}^{mf}mf^{HCN}\right)\left(V-E_{h}\right)$$

where *ms^HCN^* and *mf^HCN^* respectively defined the gating variables for the slow and fast components of the current through HCN channels, and $HCN_{ms}^{mf}$ defined the ratio of the fast to slow HCN conductance values. The activation gating particles for the slow and fast HCN components were governed by the following equations:

$$mf_{\infty }^{HCN}={\left[1+\exp\left(\frac{V+V_{mf}^{HCN}}{k_{mf}^{HCN}}\right)\right]}^{-1.36}\;ms_{\infty }^{HCN}={\left[1+\exp\left(\frac{V+V_{ms}^{HCN}}{k_{ms}^{HCN}}\right)\right]}^{-58.5}$$

$$\tau_{mf}^{HCN}=F_{mf}^{HCN}\left\{\frac{0.51}{\exp\left[\left(V-1.7\right)/10\right]+\exp\left[-\left(V+340\right)/52\right]}\right\}\;\tau_{ms}^{HCN}=F_{ms}^{HCN}\left\{\frac{5.6}{\exp\left[\left(V-17\right)/14\right]+\exp\left[-\left(V+260\right)/43\right]}\right\}.$$

#### The persistent sodium channel.

The NaP model was adopted from Dickson et al. (2000), Fransén et al. (2004), and Magistretti and Alonso (1999), and the current through this sodium channel was:

$$I_{NaP}=g^{NaP}mh\left(V-E_{Na}\right).$$

The activation gating particle was defined by:

$$m_{\infty }^{NaP}={\left\{1+\exp\left[\frac{-\left(V+V_{m}^{NaP}\right)}{k_{m}^{NaP}}\right]\right\}}^{-1}$$

$$\tau_{m}^{NaP}=F_{m}^{NaP}{\left(\left\{\frac{91\left(V+38\right)}{1-\exp\left[-\left(V+38\right)/5\right]}\right\}+\left\{\frac{-62\left(V+38\right)}{1-\exp\left[\left(V+38\right)/5\right]}\right\}\right)}^{-1}.$$

The inactivation gating particle was defined by:

$$h_{\infty }^{NaP}={\left\{1+\exp\left[\frac{\left(V+V_{h}^{NaP}\right)}{k_{h}^{NaP}}\right]\right\}}^{-1}$$

$$\tau_{h}^{NaP}=F_{h}^{NaP}{\left(\left\{\frac{-0.00288\left(V+17.049\right)}{1-\exp\left[\left(V-49.1\right)/4.63\right]}\right\}+\left\{\frac{0.00694\left(V+64.409\right)}{1-\exp\left[-\left(V+447\right)/2.63\right]}\right\}\right)}^{-1}.$$

#### The transient A-type potassium channel.

The KA model was adopted from Dudman and Nolan (2009), and the current through this potassium channel was:

$$I_{KA}=g^{KA}mh\left(V-E_{K}\right).$$

The activation gating particle was governed by the following equations:

$$m_{\infty }^{KA}={\left[1+\exp\left(\frac{V_{m}^{KA}-V}{k_{m}^{KA}}\right)\right]}^{-1}\;\tau_{m}^{KA}=F_{m}^{KA}\left(\frac{1}{\alpha_{m}^{KA}+\beta_{m}^{KA}}\right)$$

$$\alpha_{m}^{KA}=\frac{0.15\left[\left(V+18.3\right)/15\right]}{\left\{1-\exp\left[-\left(V+18.3\right)/15\right]\right\}}\;\beta_{m}^{KA}=\frac{0.15\left[\left(V+18.3\right)/-15\right]}{\left\{1-\exp\left[\left(V+18.3\right)/15\right]\right\}}.$$

The inactivation gating particle was governed by the following equations:

$$h_{\infty }^{KA}=1-{\left[1+\exp\left(\frac{V_{h}^{KA}-V}{k_{h}^{KA}}\right)\right]}^{-1}\;\tau_{h}^{KA}=F_{h}^{KA}\left(\frac{1}{\alpha_{h}^{KA}+\beta_{h}^{KA}}\right)$$

$$\alpha_{h}^{KA}=\frac{0.082\left[\left(V+58\right)/-8.2\right]}{\left\{1-\exp\left[\left(V+58\right)/8.2\right]\right\}}\;\beta_{m}^{KA}=\frac{0.082\left[\left(V+58\right)/8.2\right]}{\left\{1-\exp\left[-\left(V+8.2\right)/8.2\right]\right\}}.$$

#### The high-voltage-activated calcium channel.

The HVA calcium channel model was fitted with corresponding electrophysiological data from Bruehl and Wadman (1999) and Castelli and Magistretti (2006). The current through this channel followed GHK conventions, with the default extracellular and cytosolic calcium concentrations set at 2 mM and 100 nM, respectively. The conductance evolution of this channel was modeled as follows:

$$g\left(t\right)=g^{HVA}m^{3}h.$$

The activation and inactivation gating particles were governed by the following equations:

$$m_{\infty }^{HVA}={\left\{1+\exp\left[\frac{-\left(V_{m}^{HVA}+V\right)}{k_{m}^{HVA}}\right]\right\}}^{-1}\;\tau_{m}^{HVA}=0.92F_{m}^{HVA}$$

$$h_{\infty }^{HVA}={\left\{1+\exp\left[\frac{\left(V_{h}^{HVA}+V\right)}{k_{h}^{HVA}}\right]\right\}}^{-1}\;\tau_{h}^{HVA}=250F_{h}^{HVA}.$$

#### The low-voltage-activated calcium channel.

The LVA calcium channel model was fitted with corresponding electrophysiological data from Bruehl and Wadman (1999) and Pastoll et al. (2012). The current through this channel followed GHK conventions, with the default extracellular and cytosolic calcium concentrations set at 2 mM and 100 nM, respectively. The conductance evolution of this channel was modeled as follows:

$$g\left(t\right)=g^{LVA}m^{2}hs\left({\left[\text{Ca}\right]}_{c}\right)$$

where *m* and *h*, respectively, represented the voltage-dependent activation and inactivation gating particles, and *s*([Ca]*_c_*) governed calcium-dependent inactivation with [Ca]*_c_* (specified in mM). Their evolution was dictated by the following equations:

$$m_{\infty }^{LVA}={\left\{1+\exp\left[\frac{\left(V_{m}^{LVA}-V\right)}{k_{m}^{LVA}}\right]\right\}}^{-1}$$

$$\tau_{m}^{LVA}=F_{m}^{LVA}{\left\{\frac{-0.8967\left(V+7.88\right)}{\exp\left[-\left(V+7.88\right)/10\right]-1}+0.046\;\exp\left(\frac{-V}{22.73}\right)\right\}}^{-1}$$

$$h_{\infty }^{LVA}=1-{\left\{1+\exp\left[\frac{\left(V_{h}^{LVA}-V\right)}{k_{h}^{LVA}}\right]\right\}}^{-1}$$

$$\tau_{h}^{LVA}=1.2F_{h}^{LVA}{\left\{1.6\times 10^{-4}\;\exp\left(-\frac{V+79.5}{20}\right)+\frac{1}{1+\exp\left[-\left(V+5\right)/10\right]}\right\}}^{-1}$$

$$s\left({\left[\text{Ca}\right]}_{c}\right)=\frac{0.001}{0.001+{\left[\text{Ca}\right]}_{c}}.$$

#### The M-type potassium channel.

The KM model was adopted from Shah et al. (2008), and the current through this potassium channel was:

$$I_{KM}=g^{KM}m\left(V-E_{K}\right).$$

The activation gating particle was governed by the following equations:

$$m_{\infty }^{KM}={\left[1+\exp\left(\frac{V-V_{m}^{KM}}{k_{m}^{KM}}\right)\right]}^{-1}$$

$$\tau_{m}^{KM}=F_{m}^{KM}\left[60+\left(\frac{\exp\left[0.10584\left(\nu +42\right)\right]}{0.009\left\{1+\exp\left[0.2646\left(\nu +42\right)\right]\right\}}\right)\right].$$

### Intrinsic Measurements

We measured the resting membrane potential of the model neuron by allowing the model to settle at a steady-state potential when no current was injected for a period of 5 s. This was essential because there were several slow subthreshold conductances (especially HCN and the calcium-activated potassium channel) that contribute to resting membrane potential. We set the passive membrane potential, in the absence of any active subthreshold conductance, to be at −77 mV (Boehlen et al. 2013) and allowed the interactions among the several subthreshold conductances to set the steady-state resting membrane potential (*V*_RMP_). After this initial 5-s period of the simulation, *V*_RMP_ was computed as the mean of the membrane potential over a 1-s interval (5th to 6th second; Fig. 1*B*). We also calculated the standard deviation (SD) of membrane potential over the same 1-s period and set a validation criterion on this SD (<0.01 mV). This validation constraint ensured that the RMP was measured after attainment of steady state (which is slow, especially owing to the slow kinetics of HCN and SK channels), and that there were no membrane potential oscillations (which indicates the absence of a stable resting state) when no current was injected into the model. All intrinsic measurements reported below were always performed after this 6-s period (5 s for the transients to settle to steady state and 1 s for RMP measurement). Traces depicting these intrinsic measurements (e.g., Fig. 1, *C–I*) also represent the period after this initial 6-s duration.

To estimate Sag ratio in the model, we injected a hyperpolarizing step current of 200-pA amplitude for 1s and recorded the voltage response. Sag ratio (Sag) was computed as the membrane potential deflection achieved at steady state (*V*_SS_) during the current injection period divided by the peak deflection of the membrane potential (*V*_peak_) within the period of current injection (Fig. 1*C*). In assessing suprathreshold excitability of the model neuron, we measured the number of action potentials (AP) elicited by the neuron in response to different depolarizing step current injections spanning 500 ms. We defined the number of APs fired for 100- and 400-pA current injections as *N*_100_ (Fig. 1*D*) and *N*_400_ (Fig. 1*E*), respectively.

Input resistance (*R*_in_) was calculated from the steady-state voltage response (after 1 s of current injection) of the model neuron to subthreshold current pulses of amplitudes spanning −100 pA to 100 pA in steps of 20 pA. The steady-state voltage response was plotted against the corresponding amplitude of injected current, and the slope of a linear fit to this plot was assigned as the input resistance of the model (Fig. 1*F*). Spike amplitude (*V*_AP_) was computed from the first AP elicited during a 400-pA step current injection, and was defined as the difference between *V*_RMP_ and the peak membrane potential achieved during the AP (Fig. 1*G*).

As entorhinal stellates reside within an oscillatory network, it was essential that excitability measures be computed in a frequency-dependent manner. To do this, we computed well-established impedance-based measurements from its amplitude and phase profiles (Erchova et al. 2004; Giocomo et al. 2007; Haas and White 2002; Hu et al. 2009; Hu et al. 2002; [Bibr B82][Bibr B83]; Hutcheon and Yarom 2000; Narayanan and Johnston 2008; 2007). These profiles were computed by measuring the voltage response of the model to a chirp stimulus, a sinusoidal current stimulus with constant amplitude (40 pA peak-to-peak amplitude) with frequency linearly spanning from 0 to 15 Hz in 15 s (Fig. 1*H*). Frequency-dependent impedance, *Z*(*f*), was computed as the ratio between the Fourier transform of this voltage response and the Fourier transform of the chirp stimulus. The magnitude of the complex quantity defined the impedance amplitude profile (Fig. 1*H*):

|Z(f)|={Re[Z(f)]}2+{Im[Z(f)]}2

where Re[*Z*(*f*)] and Im[*Z*(*f*)] were the real and imaginary parts of the impedance *Z*(*f*), respectively. The frequency at which |Z(f)| reached its maximum value was considered as the resonance frequency, *f*_R_, and resonance strength (*Q*_R_) was defined as the ratio of |*Z*(*f*_R_)| to |*Z*(0.5)| (Fig. 1*H*). The impedance phase profile ϕ(*f*) was computed as:

ϕ(f)=tan−1Im[Z(f)]Re[Z(f)].

The total inductive area, Φ_L_, defined as the area under the inductive part of ϕ(*f*), was calculated based on the impedance phase profile (Narayanan and Johnston 2008):

$$\Phi_{\text{L}}=\int_{\phi \left(f\right)>0}\phi \left(f\right)df.$$

Sub- and perithreshold membrane potential oscillations (MPO) were assessed in voltage responses of the model to depolarizing pulse current injections spanning 100–300 pA in steps of 10 pA, each lasting for 5 s (Fig. 1*I*). The last 3-s period of this 5-s period was transformed to frequency domain through the Fourier transform, and the frequency at which this spectral signal had maximum magnitude was defined as the MPO frequency (*f*_MPO_). We defined the per-threshold oscillation frequency (*f*_osc_) as the *f*_MPO_ of the subthreshold voltage response proximal to the spiking threshold of the model.

### Multiparametric Multiobjective Stochastic Search Algorithm

To generate an intrinsically heterogeneous population of LII SCs and to assess if the concomitant functional expression of all 10 intrinsic measurements manifested degeneracy in terms of the specific ion channel combinations that can elicit them, we employed a multiparametric, multiobjective stochastic search (MPMOSS) algorithm (Anirudhan and Narayanan 2015; Foster et al. 1993; Goldman et al. 2001; Marder and Taylor 2011; Mukunda and Narayanan 2017; Prinz et al. 2003; Rathour and Narayanan 2014; 2012; Srikanth and Narayanan 2015). This stochastic search was performed over 55 parameters (Table 1) and jointly validated against 10 sub- and suprathreshold measurements (Fig. 1; *V*_RMP_, SD, Sag ratio, *R*_in_, *f*_R_, *Q*_R_, *f*_osc_, *N*_100_, *N*_400_, *V*_AP_) toward matching in vitro electrophysiological recordings from LII SCs (Table 2). In executing the MPMOSS algorithm, we constructed a model neuron from specific values for each of the 55 parameters, each of which was independently and randomly picked from a uniform distribution whose bounds reflected the electrophysiological variability in that parameter (Table 1). For each such randomly chosen model, which ensured that we are not biasing our parametric ranges with any constraints, all 10 intrinsic measurements were computed and were compared against their respective electrophysiological bounds (Table 2). A model that satisfied all the 10 criteria for validation was declared valid. We repeated this procedure for 50,000 randomized picks of the 55 parameters, and validated these models against the 10 measurements. As each of these 50,000 picks was independent and randomized (within their respective bounds in Table 1), each model instance was endowed with independently unique values for each of the 55 parameters. This randomization process ensured that there was no discretization of individual parameters where they are constrained to assume only specific values and that there was no cross-parametric search constraint involving relationships between different parameters.

To further corroborate the conclusions that we draw from one set of valid models derived from these 50,000 randomized picks of 55 parameters, we generated three independent sets, each with 50,000 model variants. We performed the validation procedure (involving all measurements in Table 2) on each of these three populations to obtain three independent sets of valid model populations. We statistically compared (Kruskal-Wallis test on the 3 sets, followed by pairwise Mann-Whitney tests) each intrinsic property of valid model populations across the three independent sets to ask if the valid model populations were similar across the three distinct independent sets.

### Assessment of Intrinsic and Parametric Heterogeneity in the Valid Model Population

Heterogeneities in intrinsic properties of the population of valid LII SC models and their parametric combinations were assessed using multiple metrics. In assessing intrinsic heterogeneities, the range of each intrinsic property was computed from the valid model population and compared with the electrophysiologically determined validation ranges (Table 2). In addition, pairwise correlations between the different intrinsic measurements were computed between intrinsic properties to assess relationships between different intrinsic properties across the valid model population.

To assess parametric heterogeneities and to measure the distance between models on the 55-dimensional parametric space, we employed different distance metrics on the valid model population from each of the three independent sets mentioned above. First, we employed Pearson’s correlation coefficient to compute pairwise correlations between the 55 parameters from the different valid models. Second, to measure the distances between models, we employed metrics that accounted for the widely variable ranges of the different model parameters (Table 1). The first distance metric we employed in computing the distances between models was the Euclidean distance [*d_E_*(**x**,**y**)] computed between normalized parametric vectors **x** = (*x*_1_,*x*_2_,…*x*_55_)*^T^* and **y** = (*y*_1_,*y*_2_,…*y*_55_)*^T^* of two models:

$$d_{E}\left(x,y\right)=\sqrt{\sum_{i=1}^{55}{\left(x_{i}-y_{i}\right)}^{2}}$$

where normalization was performed for each parameter individually by rescaling its respective Min–Max range (Table 1) to 0–1. Thus, $0\leq d_{E}\left(x,y\right)\leq \sqrt{55}$.

The second distance metric that we employed to compute distances between models was the Mahalanobis distance, that inherently involves the covariance matrix of the underlying parametric distribution, thereby accounting for the variance differences across the different model parameters (De Maesschalck et al. 2000; Mahalanobis 1936). The Mahalanobis distance [*d_M_*(**x**,**y**)] between parametric vectors **x** and **y** of two models was defined as:

$$d_{M}\left(x,y\right)=\sqrt{{\left(x-y\right)}^{T}\sum^{-1}\left(x-y\right)}$$

where **x** and **y** were the unnormalized vectors containing parametric values for the two models and Σ is the covariance matrix across parameters spanning the entire distribution. While the minimum value for *d_M_*(**x**,**y**) would be 0, the maximum value would depend on the specific covariance matrix. To compute the maximum *d_M_*(**x**,**y**) for each independent set, we constructed two synthetic model parametric vectors **x**_max_ and **x**_min_, with each parametric value of these models respectively set to their respective maximum and minimum possible values (Table 1). We then computed maximum value of *d_M_* between **x**_max_ and **x**_min_ for each independent set, employing the covariance matrix computed for that specific independent set. We noted that the maximum Mahalanobis distance was very similar across the three independent sets.

### Virtual Knockout Models

To assess the impact of individual channels on each of the 10 intrinsic measurements within the valid model population, we employed the virtual knockout model (VKM) approach (Anirudhan and Narayanan 2015; Mukunda and Narayanan 2017; Rathour and Narayanan 2014). In doing this, we first set the conductance value of each of the 9 active ion channels independently to zero for each of the valid models. We then computed all the 10 intrinsic measurements for each model, and assessed the sensitivity of each measurement to the different channels from the statistics of postknockout change in the measurements across all valid models. When some of the channels were knocked out, certain valid models elicited spontaneous spiking or showed depolarization-induced block (when depolarizing currents were injected). These VKMs were not included into the analysis for assessing the sensitivities, because this precluded computation of all 10 measurements from such models.

### Spike-Triggered Average and Associated Measurements

For estimation of STA, a zero-mean Gaussian white noise (GWN) with a standard deviation σ_noise_ was injected into the neuron for 1,000 s. σ_noise_ was adjusted such that overall action potential firing rate was ~1 Hz in the model under consideration. This ensured that the spikes were isolated and aperiodic, thereby establishing statistical independence of the current samples used in arriving at the STA (Agüera y Arcas and Fairhall 2003; Das and Narayanan 2015; 2014; 2017). The STA was computed from the injected current for a period of 300 ms preceding the spike and averaged over all spikes across the time period of simulation, translating to ∼1,000 spikes for each STA computation. STA kernels were smoothed using a median filter spanning a 1-ms window for representation purposes and for computing quantitative measurements that were derived from the STA.

Quantitative metrics for spectral selectivity in the STA, for coincidence detection windows and intrinsic excitability, were derived from the STA and its Fourier transform (Das and Narayanan 2015; 2014; 2017). Specifically, the frequency at which the magnitude of the Fourier transform of the STA peaked was defined as the STA characteristic frequency (*f*_STA_). STA selectivity strength (*Q*_STA_) was defined as the ratio of |STA(*f*_STA_)| to |STA(0.5)|. The peak positive current in the STA kernel was defined as $I_{STA}^{peak}$, which constitutes a measure of excitability. To quantify the window for integration/coincidence detection, we defined the spike-proximal positive lobe (SPPL) as the temporal domain that was adjacent to the spike where the STA was positive (Das and Narayanan 2015; 2017). The total coincidence detection window, CDW (*T*_TCDW_), was computed as the temporal distance from the spike location (*t* = 0 ms) to the first zero crossing in the STA. *T*_TCDW_ constitutes the entire temporal expanse over which the inputs were positively weighted and hence covered the entire temporal spread of SPPL. To account for the specific shape of the STA in defining the coincidence detection window, we defined an effective CDW (*T*_ECDW_), which was a STA-weighted measure of SPPL (Das and Narayanan 2015; 2017):

$$T_{ECDW}=\sqrt{\frac{\int_{-T_{TCDW}}^{0}t^{2}STA\left(t\right)dt}{\int_{-T_{TCDW}}^{0}STA^{2}\left(t\right)dt}}$$

### Computational Details

All simulations were performed using the NEURON programming environment (Carnevale and Hines 2006) at 34°C, with a simulation step size of 25 μs. All data analyses and plotting were executed using custom-written software within the IGOR pro (Wavemetrics) and MATLAB (Mathworks) environments. All statistical analyses were performed using the R statistical package (R Core Team 2013).

## RESULTS

The principal goal of this study was to assess the impact of heterogeneities in channel properties on cellular-scale physiological signatures of LII stellate cells of the medial entorhinal cortex. We approached this by building an unbiased stochastic search-based conductance-based population of LII SCs that satisfied several of their unique in vitro electrophysiological signatures. Apart from providing an efficacious substrate for understanding the roles of channel parameters, intrinsic measurements and associated heterogeneities in entorhinal function, our goal in building these models was threefold. First, a heterogeneous population of LII SC models that satisfied several in vitro electrophysiological constraints would provide us the means to assess if there was significant degeneracy in the emergence of these measurements, or if there was a requirement on unique mappings between channel properties and physiological measurements. Second, such a heterogeneous population would allow us to establish the specific roles of different channels in mediating or regulating different physiological properties, and assess variability in such regulatory roles. Third, and importantly, as these models match with LII SC models in several ways, they provide an ideal foundation for making quantitative predictions about stellate cell physiology, which can be electrophysiologically tested.

Toward this, we first hand-tuned a conductance-based, biophysically and physiologically relevant base model that was characterized as a single-compartmental cylindrical model with different passive and active properties (see methods). The model was endowed with 9 different active ion channels (besides leak channels), and matched with 10 distinct in vitro electrophysiological measurements obtained from LII SCs (Fig. 1, Table 2). These electrophysiological measurements included the significant sag observed in response to pulse current injections (Fig. 1*C*), theta-frequency membrane potential resonance that exhibited strong spectral selectivity (Fig. 1*H*), and importantly the robust subthreshold membrane potential oscillations in the theta-frequency range at different depolarized potentials (Fig. 1*I*). The 55 active and passive parameters that governed LII SC models, and their respective values in the base model, are listed in Table 1.

### Diverse Depolarization-Dependent Evolution of Membrane Potential Oscillations in Stochastically Generated Stellate Cell Models

We employed the base model parameters (Table 1) as the substrate for a multiparametric stochastic search algorithm for models that would meet multiple objectives in terms of matching with the in vitro electrophysiological properties of LII SCs. The range of individual parameters over which this multiparametric (55 parameters), multiobjective (10 measurements) stochastic search (MPMOSS) algorithm was executed is provided in Table 1. We generated a test population of 50,000 model cells by sampling these model parameters, and subjected these model cells to validation based on the physiologically observed range of sub- and suprathreshold measurements (Table 2). First, we found a subpopulation of these models where all measurements, except for the ability to express theta-frequency membrane potential oscillations, were within the specified bounds. We depolarized this subpopulation of models and asked if these models expressed robust subthreshold oscillations in their membrane potentials.

**Fig. 2.**
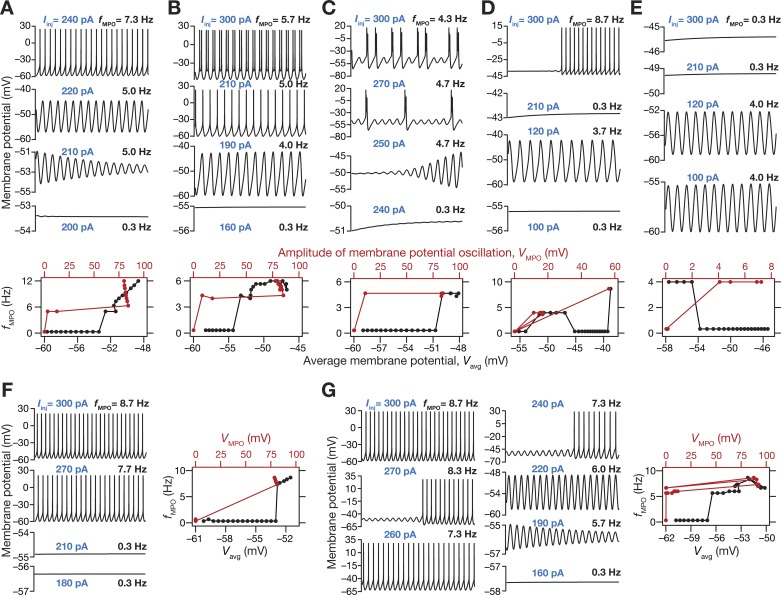
A multiparametric stochastic search algorithm yielded stellate cell models with distinct types of robust sub- and suprathreshold oscillations spanning different voltage levels. *A–E* (*top*) and *F, G* (*left*): voltage traces of model cells showing sub- and suprathreshold membrane potential oscillations (MPOs), when injected with different levels of depolarizing currents (the value of injected current used, *I*_inj_, for each voltage trace is provided in blue text). *A–E (bottom)* and *F–G* (*right*): frequency of MPOs plotted as a function of average membrane potential, *V*_avg_ (bottom axis) and MPO amplitude, *V*_MPO_ (top axis). Plots in *A–G* constitute data from different model cells, and depict representative features from distinct subpopulations of models. *A*: robust subthreshold MPOs emerge before the neuron switches to regular spiking activity that manifests when the subthreshold MPOs cross threshold. *B*: robust subthreshold MPOs emerge before the neuron abruptly switches to firing spike doublets when the subthreshold MPOs cross threshold. *C*: neuron switches to robust MPOs at perithreshold voltages, with intermittent burst spiking activity. The frequency of burst occurrence increases with increasing current injections. Such models are reminiscent of neurons exhibiting theta skipping, where spikes occur at regular intervals but not on every theta cycle. *D*: model exhibits robust theta range subthreshold oscillations, but does not directly switch to spiking behavior from MPOs with increased current injection. A range of intermittent current injections results in responses that are bereft of any MPOs. These models eventually switch to regular firing at higher current injections. *E*: same as *D* but these models do not switch to firing action potentials after exhibiting theta-range MPOs even at higher depolarization or current injections (until 300-pA depolarizing current). *F*: these models abruptly switch from firing no action potentials to regular spiking, without any intermediate phase of exhibiting subthreshold oscillations. *G*: model manifests robust subthreshold oscillations, but switches between subthreshold oscillations and regular spiking with increasing current injections. All these analyses were for current injections ranging from 100 to 300 pA for a total duration of 5 s, of which the last 3-s period is depicted and was used for further analyses.

We found that the depolarization-dependent evolution of sub- and suprathreshold (regular spiking behavior) oscillations exhibited significant diversity across different models within this subpopulation (Fig. 2). In most models within this subpopulation, consistent with corresponding experimental observations (Alonso and Llinás 1989), we observed the emergence of robust theta-range subthreshold MPOs with membrane potential approaching near spiking threshold, with further depolarization resulting in spiking activity (Fig. 2*A*) or spike doublets (Fig. 2*B*) or bursts (Fig. 2*C*) riding over subthreshold MPOs (Alonso and Klink 1993). Among these, there were some models that exhibited theta skipping (Alonso and Klink 1993), where spikes or bursts were regular, but were not observed on every cycle of the subthreshold MPO (Fig. 2*C*). In other models, incrementally higher depolarization resulted in the cell switching from rest to subthreshold MPOs to a state that was bereft of MPOs (Fig. 2, *D* and *E*). Whereas in certain models, further depolarization would result in regular spiking (Fig. 2*D*), in other models the absence of MPOs persisted with the model not entering spiking behavior until 300 pA of current injection (Fig. 2*E*). In rare cases where the model displayed spiking behavior without transitioning through subthreshold oscillations (Fig. 2*F*), the model was not included as a valid model because such models did not meet the in vitro electrophysiological constraint on theta-frequency perithreshold oscillations. Finally, a small subset of valid models manifested robust subthreshold oscillations, but switched back and forth between subthreshold oscillations and regular spiking with increasing current injections (Fig. 2*G*).

### The Stochastic Search Strategy Yielded an Intrinsically Heterogeneous Population of Models that Matched Several In Vitro Electrophysiological Signatures of Stellate Cells

**Fig. 3.**
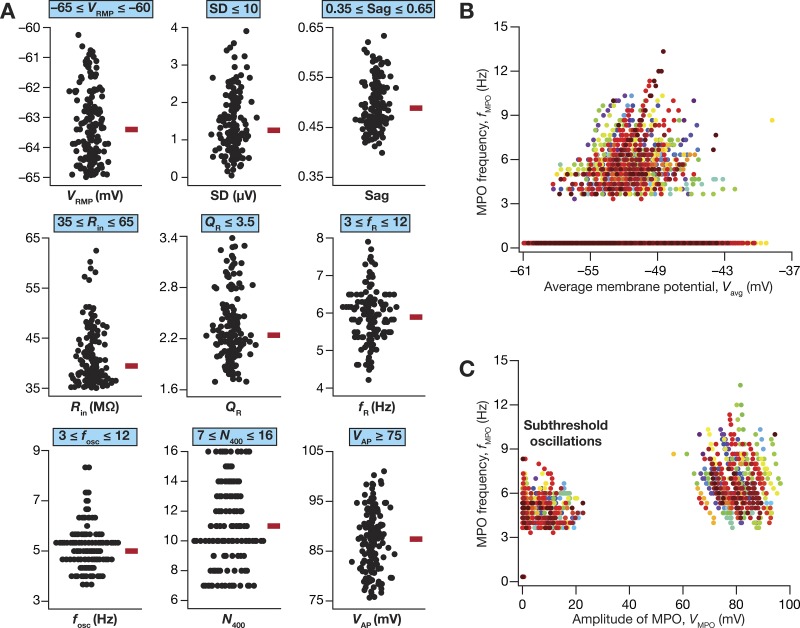
Heterogeneous distribution of physiologically relevant measurements in valid medial entorhinal cortex (MEC) layer II stellate cell models obtained after a multiparametric, multiobjective stochastic search. *A*: bee-swarm plots depicting the distribution of 9 measurements in the 155 valid models. The red rectangle adjacent to each plot depicts the respective median value. The electrophysiologically derived validation bounds for each of these measurements (Table 2) are provided above each plot, depicting that these measurements are indeed within the valid range and that they manifest heterogeneity encompassing a large span within the validity bounds. *B*: frequency of MPOs for the 155 valid models plotted as a function of average membrane potential of the oscillation, *V*_avg_. *C*: frequency of MPOs for the 155 valid models plotted as a function of MPO amplitude, *V*_MPO_. The two distinct clusters here demarcate sub- and suprathreshold oscillations, with suprathreshold oscillations corresponding to regular action potential firing. For *B* and *C*, 21 data points represent each valid model, with each data point obtained with different depolarizing current injections (*e.g*., Fig. 2). The clusters around 0 Hz in *B* and *C* correspond to voltage traces, obtained in response to some values of current injection in a given valid model, that did not manifest sub-/suprathreshold oscillations but elicited transient fluctuations (e.g., the bottom-most voltage trace in Fig. 2*A*). Each model is depicted with a unique marker.

Of 50,000 models that were generated as part of the stochastic search strategy, only 155 models (*N*_valid_ = 155) were valid when we constrained them against all the 10 in vitro electrophysiological measurements (Table 2), including their ability to manifest robust perithreshold theta-frequency oscillations. We plotted all the 10 electrophysiological measurements in this model population to assess if they were clustered around their respective base model values (Fig. 1) or if they were distributed to span the range of valid model measurements (Table 2). Whereas a clustered set of measurements would have implied a near-homogeneous population of models, a distributed pattern that spanned the range of respective in vitro electrophysiological counterparts would provide us with a heterogeneous model population that reflects experimental variability in the respective measurements. We found all measurements to spread over a large span, with most of them covering the entire min-max range of their respective bounds (Fig. 3*A*; note that *N*_100_ has not been plotted because it was required to be zero for model validity). We observed the emergence of sub- and suprathreshold membrane potential oscillations in these models when the average membrane voltage was between –59 mV and –45 mV (Fig. 3*B*). To distinguish between sub- and suprathreshold oscillations, we plotted the frequency of these membrane potential oscillations against their peak-to-peak amplitude (Fig. 3*C*). Two clearly separable clusters were observed, with all subthreshold oscillations clustered at the low-amplitude range (<25 mV), while the action potentials formed a cluster with amplitudes greater than ~60 mV. Importantly, these observations also demonstrate that the characteristics of membrane potential oscillations exhibited significant heterogeneity, thus matching the in vitro electrophysiological variability observed in LII SCs.

**Fig. 4.**
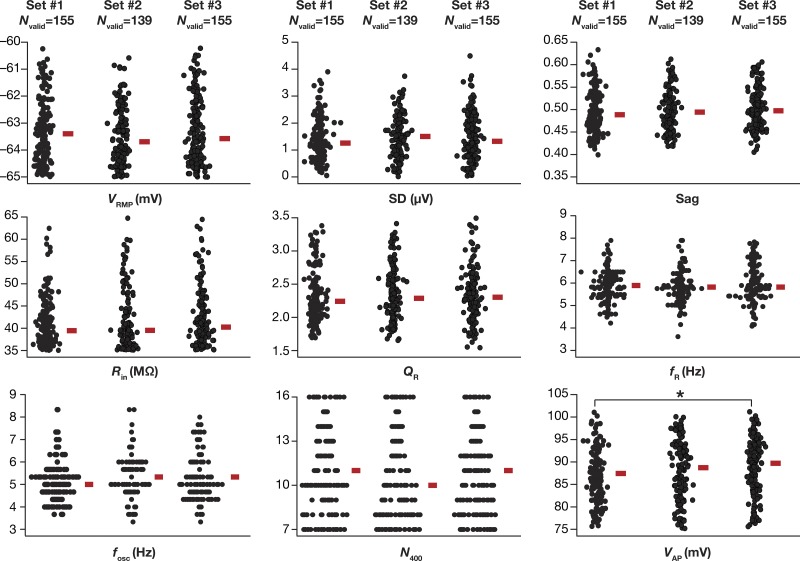
Distribution of physiologically relevant measurements in valid medial entorhinal cortex (MEC) layer II stellate cell models obtained from 3 independent sets were not significantly different. Bee-swarm plots depicting the distribution of 9 measurements in valid model populations obtained from 3 independent MPMOSS procedures. The rectangle adjacent to each plot depicts the respective median value. Each MPMOSS procedure involved 50,000 randomized picks of the 55 model parameters (Table 1), followed by a validation procedure involving the 10 intrinsic measurements (Table 2). The numbers of valid models obtained from each of the three MPMOSS procedures were 155, 139, and 155. None of the 9 intrinsic measurements depicted here were significantly different across the 3 independent sets (Kruskal-Wallis test, *P* > 0.05). Pairwise statistical comparisons of these intrinsic measurements across independent sets showed significant difference only between valid model *set 1* and valid model *set 3* for spike amplitude (*V*_AP_; *P = 0.028, Mann-Whitney test). All other measurements across all pairwise comparisons yielded *P* > 0.05, Mann-Whitney test.

Although we had spanned a large population of 50,000 independent stochastic models with nonunique parameters and their combination, it was possible that our conclusions were specific to the subpopulation of these stochastic samples. Would the intrinsic heterogeneities in the population be different if a different set of 50,000 samples were chosen in arriving at the valid models? Would the intrinsic properties that we obtain with valid model populations from such an independent set be different from those obtained with the current set of samples? To address these questions, we generated three independent sets, each with 50,000 model variants, and performed the validation procedure (involving all measurements in Table 2) on each of these three populations (Fig. 4). We found that the valid model populations obtained across the three independent runs of the MPMOSS algorithm were not significantly distinct, thereby confirming the absence of a strong sampling bias in our model generation procedures. Together, the biophysically and physiologically constrained MPMOSS algorithm yielded a population of LII SC models that manifested considerable intrinsic heterogeneities (Figs. 3 and 4) matching the ranges observed in corresponding in vitro electrophysiological measurements (Table 2).

### The Valid Model Population Manifested Cellular-Scale Degeneracy

**Fig. 5.**
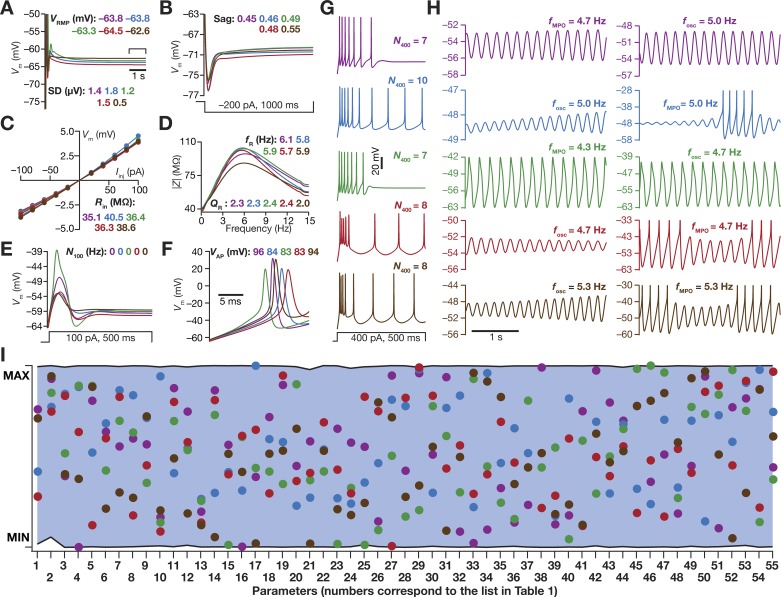
Disparate combinations of model parameters resulted in similar physiological measurements in 5 randomly chosen valid stellate cell models. *A–H*: voltage traces and 10 physiologically relevant measurements for 5 randomly chosen valid models obtained after MPMOSS. *A*: resting membrane potential (*V*_RMP_) and its standard deviation (SD). *B*: Sag ratio. *C*: input resistance (*R*_in_). *D*: resonance frequency (*f*_R_) and resonance strength (*Q*_R_). *E*: number of action potentials for a step current injection of 100 pA for 500 ms (*N*_100_). *F*: amplitude of action potential (*V*_AP_). *G*: number of action potentials for a step current injection of 400 pA for 500 ms (*N*_400_). *H*: perithreshold membrane potential oscillation frequency (*f*_osc_). *I*: normalized values of each of the 55 parameters that were employed in the generation of stellate cell models, shown for the 5 randomly chosen models depicted in *A–H*. Each parameter was normalized by the respective minimum and maximum values that bound the stochastic search for that parameter (Table 1). Parameters associated with corresponding model traces in *A–H* are depicted with identically color-coded markers in *I*. The shaded region between two black lines represents the measured min-max span for each of the 55 parameters across all valid models (not just the 5 chosen models depicted here). It may be noted that all parameters covered almost the entire stretch of their respective bounds (Table 1). The firing patterns observed in *G* are qualitatively similar to those observed in LII stellate cells (Alonso and Klink 1993; Dickson et al. 2000; Fernandez and White 2008; Khawaja et al. 2007; van der Linden and Lopes da Silva 1998) and quantitatively match the firing rates in these neurons for a 400-pA current injection (Table 2).

What were the specific constraints on the 55 different parameters in yielding the valid model population that concomitantly matched several in vitro electrophysiological signatures of stellate cells? Were these parameters clustered around specific values, thereby placing significant constraints on the different channels, their properties, and their expression profiles? Would individual channels have to be maintained at specific expression levels for models to match all 10 in vitro electrophysiological signatures? To address these questions, we first randomly picked 5 of the 155 valid models that exhibited similar measurements (Fig. 5, *A–H*), and asked if the set of parameters governing these models was also similar. We normalized each of the 55 parameters from these five models with reference to their respective min-max values, and found none of these models to follow any specific trend in their parametric values with most parameters spreading across the entire range that they were allowed to span (Fig. 5*I*). These observations provided the first line of evidence for the expression of degeneracy in stellate cell models, whereby models with very similar functional characteristics emerged from disparate parametric combinations. To confirm this across all valid models, we assessed the distributions of each parameter for all the 155 valid models, and found their spread to span the entire testing range for all parameters (Fig. 5*I*; shaded region).

**Fig. 6.**
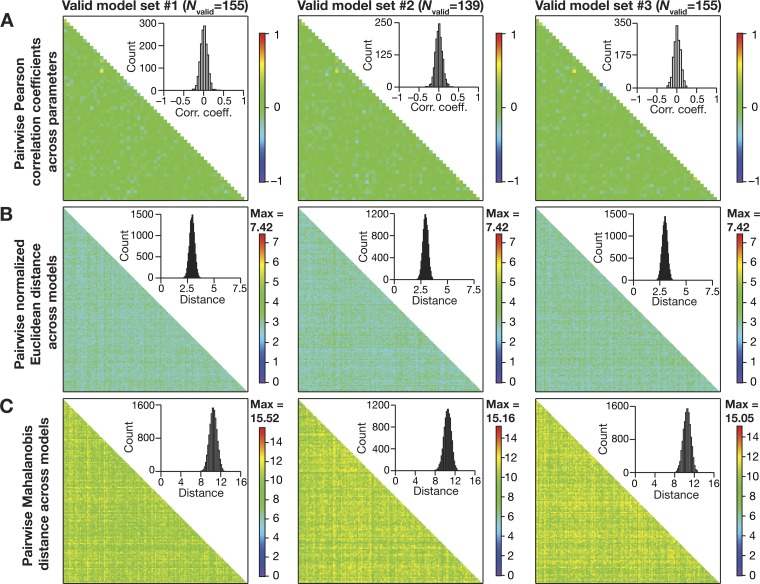
Expression of cellular-scale degeneracy in heterogeneous populations of valid stellate cell models with weak pairwise correlation among parameters. *A*: heat map of the pairwise Pearson’s correlation coefficient values between 55 parameters (Table 1) for each of the 3 valid model populations (obtained with 3 independent MPMOSS procedures). *Insets*: respective distribution of the 1,485 unique pairwise correlation coefficient values obtained across the 55 parameters. *B* and *C*: heat map of the pairwise distances between model parameters for each of the 3 valid model sets. The lower triangular part of the distance matrix is depicted, with matrix size dependent on the valid model set (*set 1*: 155 × 155; *set 2*: 139 × 139; *set 3*: 155 × 155). *Insets*: respective distribution of the unique pairwise distance values obtained across the respective valid model population. Plots in *B* and *C*, respectively, depict the pairwise normalized Euclidean and the pairwise Mahalanobis distances between model parameters.

Although the distributions of individual parameters span their respective ranges, was it essential that there are strong pairwise constraints on different parameters toward obtaining valid models? To address this question and to test if parametric combinations resulting in the heterogeneous valid population were independent of each other, we computed the pairwise Pearson’s correlation coefficient for each pairwise combination across all 55 parameters for all the 155 valid models (Fig. 6*A*), and found very weak correlations across different parameters (Fig. 6*A*). This was true for the two other valid model populations generated for Fig. 4 (Fig. 6*A*), thereby confirming the absence of selection bias in our model-generation procedures. To further assess the possibility of parametric clustering, whereby models cluster in the 55-dimensional parametric space, we assessed parametric distances between these valid models. To avoid the deleterious impact of highly variable ranges of parameters (Table 1) on distance measurements, we employed two distance metrics that explicitly accounted for this variability: the normalized Euclidean distance (Fig. 6*B*) and the Mahalanobis distance (Fig. 6*C*). With either distance measure and with all three valid model populations we found that the valid models were distal with reference to each other in the 55-dimensional parametric space. This ensured that there was no clustering of models in the parametric space, and that these were heterogeneous model populations from the perspective of parametric distances.

Together, these results demonstrated that the ability of a heterogeneous model population to concomitantly match several in vitro electrophysiological signatures of LII SCs was attainable even when individual ion channels were expressed with disparate densities and properties and when channels did not express strong pairwise correlations. These provided strong lines of evidence for the expression of cellular-scale degeneracy in LII SCs, whereby disparate combinations of channels with distinct parameters were robustly capable of eliciting similar functional characteristics.

### Virtual Knockout Models: A Many-To-Many Mapping Between Individual Channels and Physiological Characteristics Enabled the Expression of Degeneracy

A crucial requirement for the expression of such cellular-scale degeneracy is the ability of several channels to regulate different physiological characteristics (Drion et al. 2015; Rathour et al. 2016). In the absence of such capabilities, the system in essence will comprise several one-to-one mappings between channels and physiological characteristics, thereby requiring the maintenance of individual channels at specific expression levels. In asking if there was a many-to-many mapping between channels and physiological properties, we employed virtual knockouts of individual channels on all 449 models (pooled from all 3 independent sets in Fig. 4) within the heterogeneous valid model population to assess the impact of their acute removal on physiology. We employed these virtual knockout models (VKMs) to assess the impact of individual channels on all the 10 physiological measurements by calculating the change observed in each measurement after setting individual conductance values to zero (Anirudhan and Narayanan 2015; Mukunda and Narayanan 2017; Rathour and Narayanan 2014).

**Fig. 7.**
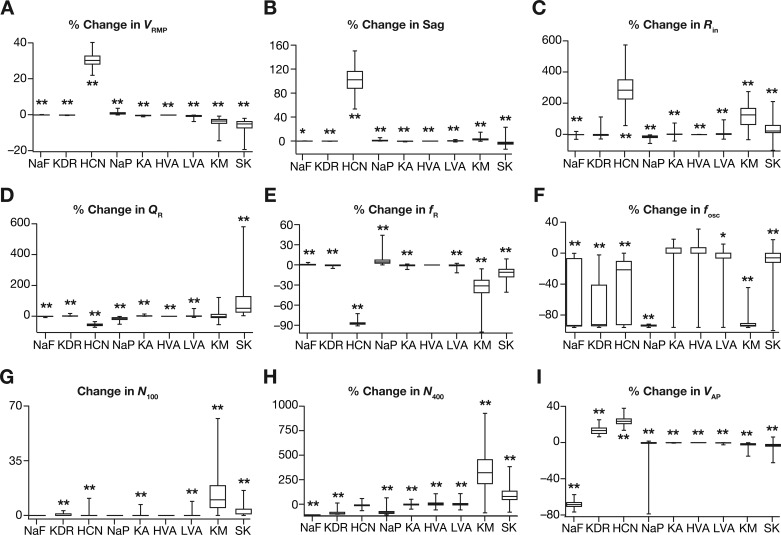
Single-channel virtual knockout models (VKMs) unveiled differential and variable dependence of measurements on individual channels. *A–I*: change in different measurement values after virtual knockout of each channel from valid models obtained from the MPMOSS algorithm. Shown are percentage changes in resting membrane potential *V*_RMP_ (*A*), sag ratio (*B*), input resistance *R*_in_ (*C*), resonance strength *Q*_R_ (*D*), resonance frequency *f*_R_ (*E*), and perithreshold oscillation frequency *f*_osc_ (*F*). Change in number of action potential elicited for 100-pA current injection (*N*_100_) is represented as a count (*G*) as *N*_100_ for all valid models was constrained to be zero, whereas changes in the number of action potentials elicited for 400-pA current injection *N*_400_ (*H*) and in action potential amplitude *V*_AP_ (*I*) are depicted as percentages. Note that VKMs that spontaneously fired or entered depolarization-induced block were removed from analyses owing to the inability to obtain subthreshold measurements. Consequently, for KDR knockouts *N*_VKM_ = 103, for KM knockouts *N*_VKM_ = 349, for SK knockouts *N*_VKM_ = 412 and all the other channel knockouts *N*_VKM_ = 449. For *A–I*, *P < 0.01, **P < 0.001, Wilcoxon rank sum test, assessing if the observed percentage changes were significantly different from a “no change” scenario.

As expected, *V*_RMP_ (Fig. 7*A*) was largely unaffected by the knockout of suprathreshold conductances (KDR, NaF, and HVA) with all subthreshold channels showing differential and heterogeneous regulation of *V*_RMP_. Specifically, consistent with prior in vitro electrophysiological recordings (Dickson et al. 2000), HCN VKMs showed large and variable hyperpolarizing shifts in *V*_RMP_ with reference to their respective base valid models. In addition, knockout of KM or SK channels resulted in variable depolarizing shifts to *V*_RMP_, but VKMs of NaP, KA, and LVA channels did not significantly alter *V*_RMP_. Although Sag ratio was expectedly (Dickson et al. 2000) reliant on HCN channels (Fig. 7*B*), there were other channels, including NaP, LVA, SK, and KM channels, which also significantly contributed to the specific value of Sag ratio. Input resistance (Fig. 7*C*) was critically altered by HCN channel knockouts (Dickson et al. 2000), with other subthreshold channels including KM and SK also showing large impacts on *R*_in_.

Resonance strength (Fig. 7*D*) and resonance frequency (Fig. 7*E*) were dramatically reduced in HCN knockouts (Boehlen et al. 2013; Erchova et al. 2004), with NaP, KM, and SK channel knockouts also showing significant changes in both measurements. Of all the assessed measurements, we found the frequency of perithreshold membrane potential oscillations to be the most sensitive measurement to channel knockouts, with most channels having a significant, yet variable, effect on *f*_osc_ (Fig. 7*F*). We observed the most reliable and least variable effect on *f*_osc_ with the removal of NaP, which consistently resulted in the loss of perithreshold oscillations across all models (Fig. 7*F*). This is consistent with several studies that have demonstrated the importance of persistent sodium channels in the emergence of perithreshold MPOs in LII SCs (Alonso and Llinás 1989; Boehlen et al. 2013; Dickson et al. 2000; Klink and Alonso 1993). Although NaP was the dominant channel that acted as the amplifying conductance (Hutcheon and Yarom 2000) in the emergence of perithreshold MPOs, we found heterogeneity across models in the specific resonating conductance that enabled these MPOs. Specifically, whereas in some models, the removal of HCN channels resulted in complete loss of MPOs, in other models the same result was achieved by the removal of KM channels. These observations suggested that the two conductances, HCN and KM, synergistically contributed as resonating conductances toward the emergence of perithreshold MPOs in stellate cells (Boehlen et al. 2013; Nolan et al. 2007). Although most of the other channels showed regulatory capabilities in terms of regulating perithreshold *f*_osc_, unlike NaP, HCN, and KM channels, their removal did not result in complete elimination of MPOs in most models. The critical dependence of *f*_osc_ on KDR removal was just a reflection of high excitability of KDR VKMs, where the cells either spontaneously fired or abruptly switched to regular spiking with small current injections resulting in the complete absence of perithreshold oscillations.

*N*_100_ was significantly higher with the deletion of KM or SK channels, with little or no effect with deletion of other channels (Fig. 7*G*). As spiking activity is critically reliant on KDR and NaF channels, their removal significantly reduced *N*_400_ (Fig. 7*H*). In addition, whereas the removal of the subthreshold regenerative conductance NaP reduced *N*_400_, knocking out the subthreshold restorative conductances, KM, SK, and KA, resulted in enhanced action potential firing that increased *N*_400_ to variable degrees (Fig. 7*H*). Although the two calcium channels mediate inward currents, their removal resulted in an increase (rather than a decrease) in *N*_400_ because of the presence of SK channels. Specifically, when either the HVA or the LVA channels was removed, the inward calcium current and cytosolic calcium concentration were lower, thereby resulting in lesser activation of SK channels and consequently leading to higher excitability (Fig. 7, *G* and *H*). Finally, AP amplitude (Fig. 7*I*) was expectedly reliant on the presence of NaF channels, while KDR and HCN also had a regulatory role in setting the value of *V*_AP_. It should be noted that *V*_AP_ is significantly dependent on *V*_RMP_, as *V*_RMP_ determines the fraction of sodium channels that are inactivated and are thereby unavailable for channel conduction. As the fraction of available sodium channels is higher with a hyperpolarized *V*_RMP_, *V*_AP_ is higher when *V*_RMP_ is hyperpolarized. As *V*_RMP_ was significantly hyperpolarized when HCN channels were knocked out (Fig. 7*A*), this implied that *V*_AP_ in HCN channel knockouts should be expected to be higher (Fig. 7*I*).

Together, analyses of all physiological measurements across valid models using VKMs of each of the nine active ion channels demonstrated the clear lack of one-to-one mappings between channels and physiological characteristics. Although there was dominance of certain channels in their ability to alter specific measurements, there were several channels that were capable of regulating each measurement and each channel regulated several measurements. Additionally, the effect of virtually knocking out individual channels on all measurements was differential for different channels and measurements, and was variable for even a given channel-measurement combination. The electrophysiological support from LII SCs for several of our conclusions, including the regulatory role of specific channels and the differential and variable dependencies of measurements on channels, with reference to blockade of individual channels is strong (Alonso and Llinás 1989; Boehlen et al. 2013; Dickson et al. 2000; Erchova et al. 2004; Garden et al. 2008; Klink and Alonso 1993; Nolan et al. 2007; Pastoll et al. 2012). These observations together point to a many-to-many configuration of the mapping between channel properties and cellular-scale physiological characteristics, a critical substrate for neurons to exhibit cellular-scale degeneracy (Figs. 3–6).

### Quantitative Predictions: Spike Initiation Dynamics of Stellate Cells Manifest Theta-Frequency Spectral Selectivity and Gamma-Band Coincidence Detection Capabilities

**Fig. 8.**
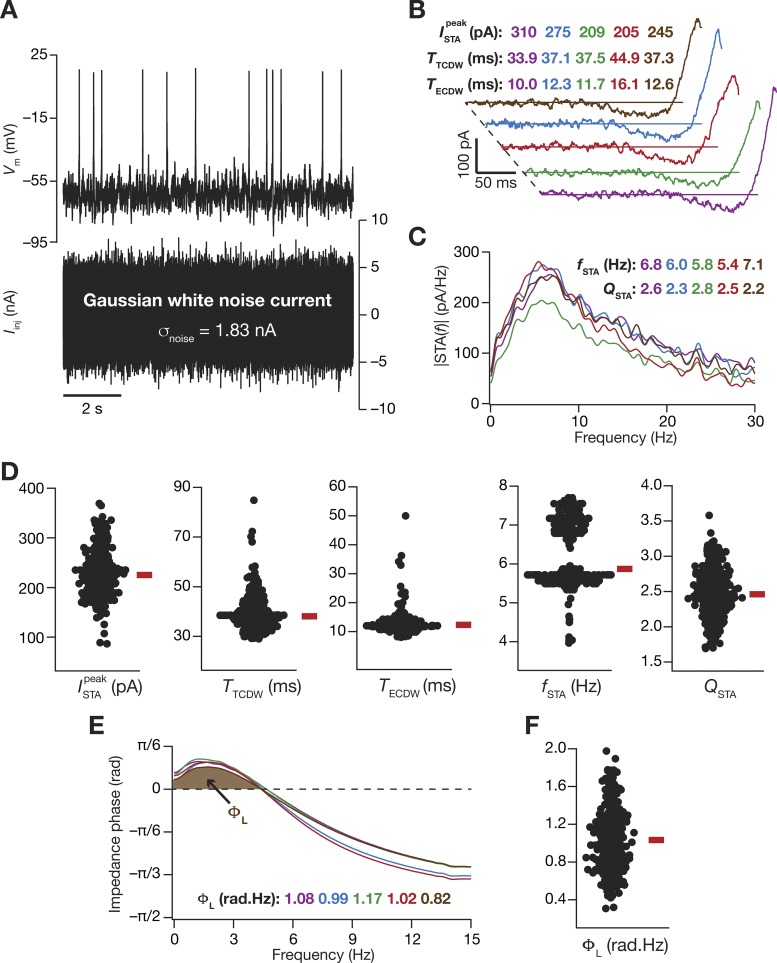
Measurements from the valid model population predict theta-frequency selectivity and gamma-range coincidence detection window in the spike-triggered average of LII stellate cells. *A*: voltage response of an example valid model (*top*) to a zero-mean Gaussian white noise (GWN) current (*bottom*) of 10-s duration. σ_noise_ = 1.83 nA. *B*: spike-triggered average (STA) of the 5 valid stellate cell models shown in Fig. 5. Measurements derived from the temporal domain representation of STA were the peak STA current $I_{STA}^{peak}$, the total coincidence detection window (CDW) *T*_TCDW_, and the effective CDW *T*_ECDW_. *C*: the magnitude of the Fourier transform of STA shown in *B*. Measurements derived from the spectral domain representation of STA were for the STA characteristic frequency *f*_STA_, and the strength of selectivity *Q*_STA_. *D*: bee-swarm plots representing the distribution of the 5 quantitative measures of the STA for all 449 valid models (pooled from all 3 independent sets in Fig. 4). *E*: impedance phase profiles, and with values of total inductive phase, Φ_L_, defined as the area under the curve for the leading impedance phase (shaded portion) for 5 selected models. Models in this figure are matched with those in Fig. 5. *F*: distribution of Φ_L_ for all 449 valid stellate cell models.

As we now had a population of models that matched with LII SC physiology, we employed these models to make specific quantitative predictions about critical physiological characteristics of LII SC. Although spectral selectivity for subthreshold inputs (Boehlen et al. 2013; Erchova et al. 2004; Garden et al. 2008; Giocomo and Hasselmo 2009; 2008; Giocomo et al. 2007; Haas and White 2002) and its relationship to spike train patterns (Engel et al. 2008) have been assessed in LII stellate cells, it is not known if such subthreshold frequency selectivity translates to the spike initiation dynamics as well (Das and Narayanan 2015; 2014; 2017; Das et al. 2017). To assess this, we employed zero-mean Gaussian white noise (GWN) with standard deviation adjusted such that the overall firing rate was ~1 Hz (Fig. 8*A*). We computed the spike-triggered average (STA) as the mean of all the current stimuli (part of the GWN current, over a 300-ms period preceding each spike) that elicited a spike response in the model under consideration. We derived five distinct measurements of excitability, spectral selectivity, and coincidence detection from the STA (Das and Narayanan 2015; 2014; 2017; Das et al. 2017), and repeated this for all the 449 valid models (pooled from all 3 independent sets in Fig. 4) obtained from MPMOSS (Fig. 4).

The STA computed from the five models shown in Fig. 5 showed characteristic class II/III excitability (Fig. 8*B*), marked by the distinct presence of negative lobes in these STA (Das and Narayanan 2015; 2014; 2017; Das et al. 2017; Ermentrout et al. 2007; Haas et al. 2007; Haas and White 2002; Ratté et al. 2013). Employing quantitative metrics from the STA (Fig. 8, *B* and *C*) for all the 449 valid models, we confirmed that these neurons were endowed with class II/III characteristics. Specifically, our analyses with the valid model population of LII SCs predict that the STA of these cells show theta-frequency spectral selectivity (Fig. 8*D*, *f*_STA_) with strong selectivity strength (Fig. 8*D*, *Q*_STA_). As class II/III excitability translates to coincidence detection capabilities in these neurons (Das and Narayanan 2015; 2014; 2017; Das et al. 2017; Ratté et al. 2013), we computed two distinct measures of coincidence detection window (CDW) from the STA. Whereas the total CDW (*T*_TCDW_) considers the temporal span of the spike-proximal positive lobe of the STA, the effective CDW (*T*_ECDW_) also accounts for the specific shape of the STA in arriving at the CDW (Das and Narayanan 2015; 2014; 2017). We computed these CDW measures for all the 449 models, and quantitatively predict that the LII SCs are endowed with gamma-range (25–150 Hz translating to 6.6–40 ms) coincidence detection capabilities (inferred from the values of *T*_ECDW_; Fig. 8*D*). Finally, although it is known that the impedance phase of LII SCs manifests a low-frequency inductive lead (Erchova et al. 2004), this inductive phase lead has not been systematically quantified. To do this, we computed the total inductive phase metric (Φ_L_; Fig. 8*E*) developed in Narayanan and Johnston (2008) for each of the 449 valid models, and quantitatively predict a prominent inductive phase lead in LII SCs (Fig. 8*F*) with specific values for Φ_L_.

### Pairwise Correlations Among Measurements

**Fig. 9.**
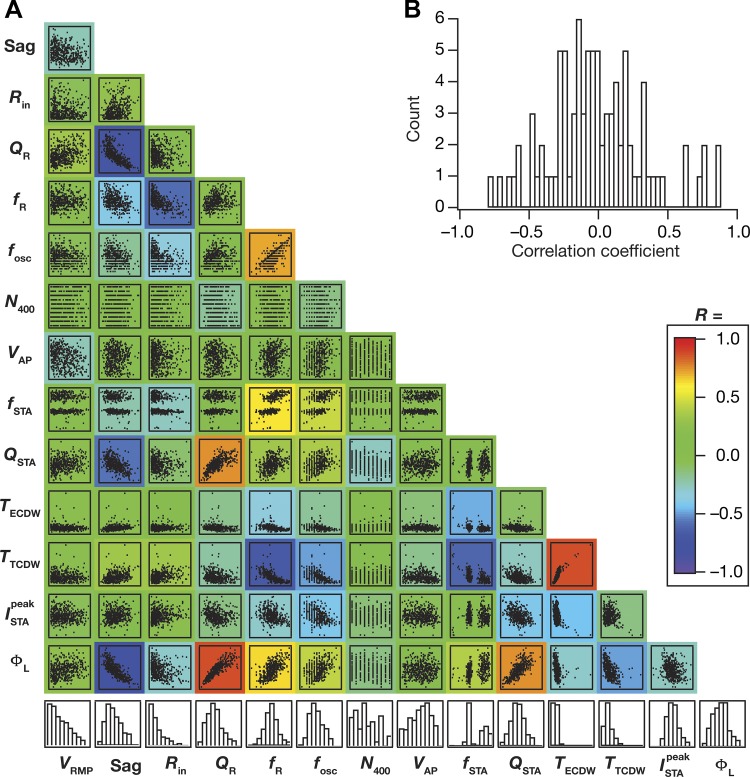
Pairwise correlations across physiological measurements from all valid stellate cell models were variable. *A*: matrix depicts the pairwise scatterplots (spanning all 449 valid models) between the 14 measurements (8 physiologically relevant measurements, namely *V*_RMP_, Sag ratio, *R*_in_, *Q*_R_, *f*_R_, *f*_osc_, *N*_400_, *V*_AP_, in Fig. 1 and the 6 predicted measurements, namely $I_{STA}^{peak}$, *T*_ECDW_, *T*_TCDW_, *f*_STA_, *Q*_STA_, Φ_L_, in Fig. 8). Histograms in the bottom row depict the span of the corresponding measurement with reference to their respective min-max ranges. Individual scatterplots are overlaid on a heat map that depicts the pairwise correlation coefficient computed for that scatterplot. *B*: distribution of the 91 unique correlation coefficient values from scatterplots in *A*.

Finally, as our analyses (Fig. 7) demonstrated a many-to-many mapping between biophysical parameters and physiological measurements, we asked if there were significant correlations among the distinct measurements. Strong correlations across these measurements (which are reflective of distinct physiological characteristics) would imply that they could be mapped to a smaller set of “core” measurements with the other measurements relegated to redundant and correlated reflections of these core measurements. In addition, strong correlations across measurements would also imply that there are measurements that are strongly reliant on the expression and properties of one specific channel (especially given the lack of significant correlations across channels conductances, Fig. 6*A*). To assess correlations across measurements, we plotted the pairwise scatterplots (Fig. 9*A*) spanning all 14 measurements (8 measurements from Fig. 1: *V*_RMP_, Sag, *R*_in_, *Q*_R_, *f*_R_, *f*_osc_, *N*_400_, *V*_AP;_ and 6 predicted measurements from Fig. 8: $I_{STA}^{peak}$, *T*_ECDW_, *T*_TCDW_, *f*_STA_, *Q*_STA_, Φ_L_) computed on the 449 valid models (pooled from all 3 independent sets in Fig. 4). We computed the Pearson’s correlation coefficient for each scatterplot and analyzed the correlation coefficients across measurements (Fig. 9, *A* and *B*). Although there were strong correlations across some measurements, a majority of these pairwise correlations were weak (Fig. 9*B*). Measurements that showed strong pairwise correlations were those that were known to be critically reliant on specific ion channels. For instance, Sag ratio showed strong negative correlation with Φ_L_, *Q*_STA_, and *Q*_R_, whereas *f*_R_ manifested strong negative correlation with *R*_in_ and *T*_TCDW_, where all these measurements are known to have a strong dependence on HCN channels (Fig. 7). However, even within this subset of measurements that were strongly dependent on one channel, we noted that only a subset of the pairwise correlations was high. For instance, with reference to the specific examples discussed above, *f*_R_ and *Q*_R_, both critically dependent on HCN channels and both derived from the impedance amplitude profile, do not show strong pairwise correlation (Fig. 9*A*).

Among measurements that defined spectral selectivity (*f*_STA_, *f*_R_, *Q*_STA_, *Q*_R_) and subthreshold oscillations (*f*_osc_), there were relatively strong correlations only between *f*_osc_*-f*_R_ and *Q*_STA_-*Q*_R_ pairs. The relatively weak correlation between sub- (*f*_R_) and suprathreshold (*f*_STA_) spectral selectivity measurements is consistent with similar conclusions involving hippocampal pyramidal neurons, where these selectivity measurements have been shown to have differential parametric dependencies (Das and Narayanan 2015; 2014; 2017; Das et al. 2017). In addition to extending such dissociation between *f*_STA_ and *f*_R_ to LII SCs, our analyses revealed weak correlation between spectral selectivity in the STA (*f*_STA_) and the perithreshold oscillatory frequency (*f*_osc_), while conforming with experimental findings (Giocomo et al. 2007; Hutcheon and Yarom 2000) on strong correlations between *f*_osc_ and *f*_R_ (Fig. 9*A*).

In summary, although there was a small percentage of measurements that showed strong pairwise correlations, a majority of their pairwise correlations were weak, further emphasizing the absence of one-to-one relationships between channels and measurements (Fig. 7).

## DISCUSSION

The prime conclusion of this study is that LII stellate cells of the medial entorhinal cortex express cellular-scale degeneracy in the concomitant expression of several unique physiological characteristics of these neurons. We arrived at this conclusion through an unbiased stochastic search algorithm that spanned 55 parameters associated with biophysically constrained models of active and passive stellate cell components. We validated the outcomes of these stochastically generated models against 10 different physiological characteristics of stellate cells. This validation process provided us with a heterogeneous population of stellate cells that exhibited cellular-scale degeneracy with weak pairwise correlations across parameters that governed these models. We employed these models to demonstrate the differential and variable dependencies of measurements on underlying channels, and also showed that the mapping between channels and measurements was many-to-many. Finally, we employed this electrophysiologically validated model population to make specific quantitative predictions that point to theta-frequency spectral selectivity and gamma-range coincidence detection capabilities in class II/III spike-triggered average of LII SCs.

### Correlations Between Electrophysiological Signatures of Distal Dendrites in CA1 Pyramidal Neurons and LII MEC Stellate Cells: Instances of Cellular-Scale Efficient Encoding?

A cursory glance at the in vitro electrophysiological properties of distal dendrites of CA1 pyramidal neurons and LII MEC stellate cells presents significant correlations between some physiological characteristics of these two structures. Although superficial layers of the MEC project to the distal dendrites of CA1 pyramidal neurons, it is LIII, and not LII, principal neurons of the MEC that project to CA1 pyramidal neurons (Andersen et al. 2006). Despite this, there are several electrophysiological characteristics of LII MEC stellates that match with the distal dendrites of CA1 pyramidal neurons. Several of these similarities are strongly related to the heavy expression profile of HCN channels in both these structures. Whereas the gradient in HCN channel density in CA1 pyramidal neurons implies a significantly sharp increase in HCN channels in the distal dendrites of CA1 pyramidal neurons (Lörincz et al. 2002; Magee 1998), the heavy expression of HCN channels in the cell body of LII MEC stellates is also well established (Boehlen et al. 2013; Dickson et al. 2000; Erchova et al. 2004; Garden et al. 2008; Giocomo and Hasselmo 2009; 2008; Giocomo et al. 2011a; Giocomo et al. 2007; Klink and Alonso 1993; Nolan et al. 2007).

As a consequence of this, these two structures are endowed with significant sag, similar input resistances, and comparable theta-band suthreshold resonance frequencies (Erchova et al. 2004; Narayanan and Johnston 2007; Pastoll et al. 2012). In addition to these, our study predicts that the STA of LII SCs should be endowed with theta-frequency spectral selectivity and gamma-band coincidence detection windows. Although it is known that the STA of LII SCs manifest class II/III excitability with coincidence detection characteristics (Haas et al. 2007; Haas and White 2002), quantification of spectral selectivity in the STA or a systematic assessment of the specific frequency band of coincidence detection capabilities has not been assessed. Our study specifically predicts the coincidence detection window (Fig. 8*D*; *T*_ECDW_) associated with the STA of LII SCs to be in the fast-gamma frequency (60–120 Hz) range, with a high theta-range spectral selectivity in the STA (Fig. 8*D*; *f*_STA_). Interestingly, quantitative predictions for these measurements for the distal dendritic region of CA1 pyramidal neurons also fall within the same spectral bands (Das and Narayanan 2015).

This confluence of STA measurements of distal dendrites in CA1 pyramidal neurons and of LII SC soma, especially that of the coincidence detection window falling within the fast gamma-frequency band, is striking from the perspective of gamma-band multiplexing that has been reported in the CA1 subregion (Colgin et al. 2009; Colgin and Moser 2010; Fernández-Ruiz et al. 2017). Specifically, gamma oscillations in the CA1 subregion have been shown to manifest stratification into distinct fast- and slow-frequency components, impinging, respectively, on distal and proximal dendritic regions. This spatially stratified frequency-division multiplexing allows for differential coupling of CA1 pyramidal neurons to afferent inputs from the MEC and CA3 through different gamma bands. Juxtaposed against this, and within the efficient coding framework (Barlow 1961; Bell and Sejnowski 1997; Lewicki 2002; Simoncelli 2003; Simoncelli and Olshausen 2001) where the response filters in a single neuron should match the natural statistics of afferent network activity (Narayanan and Johnston 2012), it may be argued that different dendritic locations should be equipped with filters (STA kernels) that are matched to the afferent inputs (different gamma frequencies) that are received by that specific location. As a specific instance of such location-dependent efficient encoding of afferent network statistics in hippocampal pyramidal neurons, it has been quantitatively postulated that the distal dendrites of CA1 pyramidal neurons are endowed with coincidence-detection windows specific to fast-gamma frequencies, whereas those of the proximal dendrites matched with slow-gamma frequencies (Das and Narayanan 2015).

In this context and given the several lines of evidence for the dominance of fast gamma oscillations in the superficial layers of MEC (Colgin 2016; Colgin et al. 2009; Trimper et al. 2017), our predictions that the CDW for stellate neurons should be in the fast-gamma band point to a similar form of efficient encoding schema in the MEC. Specifically, if the fast gamma oscillations are statistically the most prevalent in the superficial layers of MEC, it is imperative that neurons there are equipped with the machinery that is capable of detecting and processing afferent synchronous inputs that might be coincident within this frequency range. For instance, if gamma-frequency cell assemblies were to project onto these neurons (Buzsáki 2010), the detection of coincident arrival of these inputs would be infeasible if the neuron were a class I integrator endowed with an integration time constant of tens of milliseconds, but would require a class II/III coincidence detector capable of detecting gamma-frequency coincident inputs (Das and Narayanan 2015; 2017; Das et al. 2017; Ratté et al. 2013; Softky 1994). Additionally, from the efficient coding perspective, as neurons tune their response properties to efficiently represent the statistics of afferent inputs (Bell and Sejnowski 1997; de Villers-Sidani et al. 2007; deCharms et al. 1998; Hirsch and Spinelli 1970; Kilgard and Merzenich 1998; Kilgard et al. 2001; Lewicki 2002; Sharma et al. 2000; Simoncelli 2003; Simoncelli and Olshausen 2001; Stemmler and Koch 1999; Wiesel and Hubel 1963a,b), it is essential that the response properties of LII MEC neurons are tuned to the fast gamma frequency range. Thus our prediction on a fast gamma-band CDW in the STA of LII EC cells suggests the possibility of efficient encoding spanning the hippocampal formation, whereby the neuronal properties in terms of their class of excitability and specific band of frequency where their coincidence detection windows lie match with the type of gamma-frequency band that is most prevalent in that subregion (or strata in case of the CA1). A direct test of this experimental prediction would be to measure the CDW of pyramidal neurons in the CA3, of different dendritic subregions of the CA1 and of LII MEC stellates and ask if these CDW match with the respective gamma-band inputs that are prevalent in these subregions. The presence of such sharp coincidence-detection windows could also have important consequences for the precision of theta-phase locking in these neurons in terms of reducing variability of the phase of spikes with reference to an extracellular theta oscillation, thereby enhancing spike phase coherence in LII SCs (Sinha and Narayanan 2015).

Finally, encoding schema are state-dependent processes that are critically reliant on behavioral state and consequent changes in afferent activity, neuromodulatory tones, and activity-dependent plasticity (Bargmann and Marder 2013; Das et al. 2017; Denève et al. 2017; Gallistel 2017; Marder 2012; Narayanan and Johnston 2012; Ratté et al. 2013; Srikanth and Narayanan 2015). Therefore, it is important that postulates on efficient codes and relationships between neuronal activity and afferent statistics are assessed in a manner that accounts for adaptability of coding within the neuron and across the network. Such activity-dependent plasticity and neuromodulation of intrinsic properties, especially of signature characteristics such as the membrane potential oscillations, could be systematically assessed in entorhinal stellates. Specifically, as activity-dependent plasticity of several ion channels is well established across different brain regions (Johnston and Narayanan 2008; Magee and Johnston 2005; Narayanan et al. 2010; Narayanan and Johnston 2012; 2008; 2007; Shah et al. 2010; Sjöström et al. 2008), it would be important to ask if membrane potential oscillations, spectral selectivity characteristics (*f*_R_, *Q*_R_, *f*_STA_, *Q*_STA_, Φ_L_), and coincidence-detection windows are amenable to such activity-dependent plasticity that target different ion channels (Fig. 7).

### Implications of Cellular-Scale Degeneracy in LII Stellate Cell Physiology

Degeneracy, the ability of disparate structural components to elicit similar function, is a ubiquitous biological phenomenon with strong links to robust physiology and evolution (Edelman and Gally 2001; Leonardo 2005; Price and Friston 2002; Whitacre and Bender 2010; Whitacre 2010). Several studies spanning different systems have now demonstrated the expression of degeneracy, at different scales of analysis in neural systems as well (Drion et al. 2015; Marder 2011; Marder and Goaillard 2006; Marder and Prinz 2002; Marder and Taylor 2011; O’Leary and Marder 2014; O’Leary et al. 2014; Prinz et al. 2004; Vogelstein et al. 2014). Within the hippocampal formation, recent studies have demonstrated the expression of degeneracy in single-neuron electrophysiology (Mishra and Narayanan 2017; Rathour and Narayanan 2012; Srikanth and Narayanan 2015), intraneuronal functional maps (Rathour et al. 2016; Rathour and Narayanan 2014), synaptic localization required for sharp-tuning of hippocampal place fields (Basak and Narayanan 2018), short- (Mukunda and Narayanan 2017) and long-term (Anirudhan and Narayanan 2015) synaptic plasticity profiles, and network-scale response decorrelation (Mishra and Narayanan 2017). In this study, we have demonstrated the expression of cellular-scale degeneracy in LII SCs, which are endowed with unique in vitro electrophysiological signatures including the prominent theta-frequency subthreshold membrane potential oscillations.

The implications for the expression of such cellular-scale degeneracy are several. First, the many-to-many mapping between channels and physiological characteristics (Fig. 7) and the consequent degeneracy in concomitantly achieving all signature electrophysiological characteristics implies that there is no explicit necessity for maintaining individual channels at specific levels or for maintaining paired expression between channel combinations (Figs. 5 and 6). This provides several significant degrees of freedom to the protein localization and targeting machinery in achieving specific functions or in maintaining homeostasis in these functional characteristics. Second, this also implies that adaptability to external stimuli, in terms of achieving efficient codes of afferent stimuli or in encoding features of a novel stimulus structure, could be achieved through disparate combinations of plasticity in several constituent components. For instance, our results predict that the ability to achieve fast gamma-band coincidence-detection capabilities could be achieved through distinct combinations of channel parameters (Figs. 5, 6, and 8). Experimental analyses of such degeneracy in achieving efficient matching of neuronal response characteristics with the statistics of oscillatory patterns, through the use of distinct pharmacological agents that target different channels, would demonstrate the ability of different channels to regulate such efficient encoding (Das et al. 2017). Finally, degeneracy in the expression of excitability properties also implies that similar long-term plasticity profiles in these neurons could be achieved with disparate combinations of parameters. Specifically, several forms of neuronal plasticity are critically reliant on the amplitude and kinetics of cytosolic calcium entry, which in turn are dependent on neuronal excitability properties. As similar excitability profiles could be achieved with distinct combinations of constituent components, it stands to reason that similar plasticity profiles could be achieved through disparate parameter combinations (Anirudhan and Narayanan 2015; Ashhad and Narayanan 2013; Narayanan and Johnston 2010).

### Limitations of the Analyses and Future Directions

A critical future direction for the study presented here is the incorporation of biological heterogeneities into entorhinal network models that assess grid cell formation. Most models for grid cell formation are simple rate-based models that are made of homogeneous repeating units, and even models that incorporate conductance-based neurons for grid cell modeling do not account for the several biological heterogeneities that are expressed in the entorhinal network. This lacuna is especially striking because such analysis is critical for the elucidation of the mechanistic bases for grid cell formation (in terms of the channels and receptors involved) and for the quantitative understanding of the ability of the entorhinal network to elicit robust grid cell behavior in the presence of several network heterogeneities. For instance, are the several forms of network heterogeneities (i.e., intrinsic, local synaptic, and afferent connectivity) and interactions among them aiding or hampering the robustness of grid cell emergence? Are the different models for grid cell emergence robust to significant variability in channels, synapses, and afferent connectivity? How are the different signature electrophysiological characteristics of the entorhinal neurons critical in the formation of grid cells? Does class II/III excitability of LII stellate cells play a critical role in entorhinal function and in grid cell formation?

At the cellular scale, although our study incorporates several electrophysiologically characterized channels into the model, an important limitation is that the analyses is not complete in terms of all the channels that are known to be expressed in LII MEC stellates. Specifically, future models could incorporate resurgent sodium channels (Hargus et al. 2011), other calcium-activated potassium channels (Khawaja et al. 2007), and the characteristic calcium-sensitive cation-nonspecific current (Magistretti et al. 2004; Shalinsky et al. 2002). We postulate that the incorporation of these channels would augment the cellular-scale degeneracy that is reported in this present study.

A further limitation in our analysis is that the ion channel models incorporated into our neuronal models match only the ensemble dynamics of their biological counterparts. Models involving ensemble dynamics of ion channel physiology have been very helpful in assessing and understanding neuronal physiology (including oscillatory behavior) and cellular-scale degeneracy (Anirudhan and Narayanan 2015; Foster et al. 1993; Goaillard et al. 2009; Goldman et al. 2001; Golowasch et al. 2002; Hodgkin and Huxley 1952; Marder and Goaillard 2006; Marder and Taylor 2011; Mishra and Narayanan 2017; Mukunda and Narayanan 2017; Prinz et al. 2004; Rathour and Narayanan 2014; 2012; Srikanth and Narayanan 2015; Taylor et al. 2009), and we have demonstrated here that models with robust signature in vitro electrophysiological characteristics (especially robust mixed-mode membrane potential oscillations spanning different voltage depolarizations) are attainable with these deterministic model formulations.

However, if the impact of stochastic channel gating and associated channel noise were to be assessed with reference to the emergence of physiological characteristics, including that of perithreshold oscillations, it is of utmost importance to construct neuronal models that account for stochastic perturbations (Dorval and White 2005; Dudman and Nolan 2009; Rotstein et al. 2006; White et al. 1998). Future models could assess cellular-scale degeneracy in neuronal models endowed with stochastic channels, especially addressing questions about the emergence of perithreshold oscillations and the role of stochastic fluctuations within such a framework of degeneracy. Specifically, whereas noise is not considered as an essential component in the emergence of the bifurcations that result in mixed-mode oscillations (Fransén et al. 2004; Rotstein 2017; Rotstein et al. 2006; Rotstein et al. 2008), the presence of channel noise or synaptic noise could enhance the robustness of the perithreshold oscillations or make the oscillations to be noisy sinusoids (Fransén et al. 2004; Rotstein et al. 2006; White et al. 1998), outcomes that are consistent with the stochastic bifurcation structures (Bashkirtseva and Ryashko 2011; Berglund and Landon 2012; Gong and Xu 2001; Hasegawa 2004; Kanamaru et al. 2001; Kember et al. 2001; Krupa and Touboul 2016; Lindner and Schimansky-Geier 1999; Scholl et al. 2005; Tanabe and Pakdaman 2001; Tuckwell 2008; Tuckwell and Rodriguez 1998; Tuckwell et al. 2003).

A potential direction for the future would be to assess the impact of ion channel degeneracy on introduction of channel noise (introduced either as stochastic perturbations to channel kinetics or with stochastic channel models that precisely model the gating kinetics of each channel) or synaptic noise (introduced as high-conductance states or as additive noise with a specific color) into entorhinal stellate models showing mixed-mode oscillations. Although the introduction of stochastic perturbations are expected to render perithreshold oscillations to be noisy sinusoids, the specific impact of channel degeneracy on stochastic bifurcations that are resultant from parametric (of specific channels with different kinetics) and external (synaptic, through high-conductance states for instance) perturbation could shed light on gradients and heterogeneities of stellate physiology. Additionally, introduction of high-conductance states into in vitro models will allow for the replication of electrophysiological properties observed in vivo, especially in terms of reduced excitability and alterations to perithreshold oscillatory behavior that emerge as consequences of interactions of high-conductance states with individual channels (Destexhe et al. 2003; Mishra and Narayanan 2015; Schmidt-Hieber and Häusser 2013).

Future studies could build heterogeneous models to account for the signature continuum of intrinsic physiological characteristics along the dorsoventral axis of the MEC, also accounting for specific differences in morphology and channel expression that are known to change along this axis (Garden et al. 2008; Giocomo and Hasselmo 2009; 2008; Giocomo et al. 2007; Yoshida et al. 2011). Such studies will provide quantitative bases for exploring the expression of degeneracy in maintaining the dorsoventral gradients, and could be incorporated into network models for grid formation in assessing the relationship between grid-cell characteristic and neuronal intrinsic properties.

The heterogeneous model population built in this study comprises a simple single-compartmental structure that did not account for dendritic arborization or morphological heterogeneity of LII SCs. Incorporation of these into neuronal models is important owing to the critical roles that dendritic morphology and active dendritic conductances therein have been shown to play across several neuronal subtypes, including LII SCs (Cannon et al. 2010; Dhupia et al. 2015; Garden et al. 2008; Jiang et al. 2008; Mainen and Sejnowski 1996; Narayanan and Chattarji 2010; Narayanan and Johnston 2012; Pastoll et al. 2012; Schaefer et al. 2003; Schmidt-Hieber et al. 2017; Sjöström et al. 2008; Stuart and Spruston 2015; Vetter et al. 2001). Electrophysiologically, the absence of systematic cell-attached recordings of specific channels and their properties in the stellate cells has been a significant impediment in building morphologically realistic models. While future experimental studies could focus on recording channels and channel properties along the nonplanar dendritic arbor of stellate cells, future computational studies could incorporate these channels into morphologically realistic models to assess the specific roles of dendritic channels and morphological heterogeneity in grid cell formation (Schmidt-Hieber et al. 2017).

Our study provides specific quantitative predictions about the STA of LII SCs and also presents an array of cross-dependencies of measurements on different channel types. Future electrophysiological studies could systematically test these predictions, and assess efficient encoding in these structures apart from adding further evidence for the many-to-many mapping between channels and physiological characteristics. For instance, an important prediction from VKMs is on the critical role of SK channels in regulating several intrinsic measurements including membrane potential oscillations (Fig. 7). Although the expression of calcium-dependent potassium channels is established in stellate cells (Khawaja et al. 2007; Pastoll et al. 2012), the specific role of these channels in regulating resonance, impedance phase, intrinsic excitability, and membrane potential oscillations could be tested electrophysiologically using pharmacological blockers of SK channels.

## GRANTS

This work was supported by the Wellcome Trust-DBT India Alliance (Senior fellowship to R. Narayanan; IA/S/16/2/502727), the Department of Biotechnology (R. Narayanan), and the Ministry of Human Resource Development (D. Mittal, R. Narayanan).

## DISCLOSURES

No conflicts of interest, financial or otherwise, are declared by the authors.

## AUTHOR CONTRIBUTIONS

D.M. and R.N. conceived and designed research; D.M. performed experiments; D.M. analyzed data; D.M. and R.N. interpreted results of experiments; D.M. prepared figures; D.M. and R.N. drafted manuscript; D.M. and R.N. edited and revised manuscript; D.M. and R.N. approved final version of manuscript.
